# Pangenome reconstruction of *Lactobacillaceae* metabolism predicts species-specific metabolic traits

**DOI:** 10.1128/msystems.00156-24

**Published:** 2024-06-26

**Authors:** O. Ardalani, P. V. Phaneuf, O. S. Mohite, L. K. Nielsen, B. O. Palsson

**Affiliations:** 1 Novo Nordisk Foundation Center for Biosustainability, Technical University of Denmark, Lyngby, Denmark; 2 Australian Institute for Bioengineering and Nanotechnology, The University of Queensland, Brisbane, Queensland, Australia; 3 Bioinformatics and Systems Biology Program, University of California, San Diego, La Jolla, California, USA; 4 Department of Pediatrics, University of California, San Diego, La Jolla, California, USA; 5 Center for Microbiome Innovation, University of California, San Diego, La Jolla, California, USA; 6 Department of Bioengineering, University of California, San Diego, La Jolla, California, USA; Katholieke Universiteit Leuven, Leuven, Belgium

**Keywords:** *Lactobacillaceae*, genome-scale metabolic model, pangenome, genome-scale reconstruction

## Abstract

Strains across the *Lactobacillaceae* family form the basis for a trillion-dollar industry. Our understanding of the genomic basis for their key traits is fragmented, however, including the metabolism that is foundational to their industrial uses. Pangenome analysis of publicly available *Lactobacillaceae* genomes allowed us to generate genome-scale metabolic network reconstructions for 26 species of industrial importance. Their manual curation led to more than 75,000 gene-protein-reaction associations that were deployed to generate 2,446 genome-scale metabolic models. Cross-referencing genomes and known metabolic traits allowed for manual metabolic network curation and validation of the metabolic models. As a result, we provide the first pangenomic basis for metabolism in the *Lactobacillaceae* family and a collection of predictive computational metabolic models that enable a variety of practical uses.

**IMPORTANCE:**

*Lactobacillaceae*, a bacterial family foundational to a trillion-dollar industry, is increasingly relevant to biosustainability initiatives. Our study, leveraging approximately 2,400 genome sequences, provides a pangenomic analysis of *Lactobacillaceae* metabolism, creating over 2,400 curated and validated genome-scale models (GEMs). These GEMs successfully predict (i) unique, species-specific metabolic reactions; (ii) niche-enriched reactions that increase organism fitness; (iii) essential media components, offering insights into the global amino acid essentiality of *Lactobacillaceae*; and (iv) fermentation capabilities across the family, shedding light on the metabolic basis of *Lactobacillaceae*-based commercial products. This quantitative understanding of *Lactobacillaceae* metabolic properties and their genomic basis will have profound implications for the food industry and biosustainability, offering new insights and tools for strain selection and manipulation.

## INTRODUCTION

*Lactobacillaceae* are an essential family of highly diverse lactic acid bacteria. It comprises a large number of species that populate a variety of habitats (1). Due to numerous applications in food and pharmaceutical industries, *Lactobacillaceae*-dependent products have a trillion-dollar market size, including dairy, wine, probiotics, and numerous satellite industries (2–4) (see Table S1 for details), indicating their importance in microbial biotechnology and related industries.

Cost-effective DNA sequencing has led to a steadily increasing number of *Lactobacillaceae* genomes deposited in the NCBI database (5). The availability of these genomes has enabled pangenomic studies (1, 6–8). Metabolism is foundational to the industrial uses of *Lactobacillaceae,* and the generation of predictive genome-scale metabolic models (GEMs) constitutes a significant advancement in bioprocess engineering (9). GEMs are based on annotated sequences and use algorithms to forecast cellular behavior and metabolic fluxes under conditions of interest. GEMs have been shown to predict optimal growth conditions and gene manipulation targets to increase product yield by integrating complex metabolic pathways. The availability of a large number of *Lactobacillaceae* genome sequences enables pangenome-based metabolic reconstruction to create a comprehensive and predictive set of GEMs.

Despite the large market size, undeniable contribution to today’s humankind lifestyle, and availability of large sets of genomes and other omics data, only a few genome-scale models have been reconstructed for *Lactobacillaceae* members so far. Previous reconstruction efforts were limited to several model strains across the whole family, including *Lactobacillus plantarum* (10), *Lactobacillus reuteri* (11), *Lactobacillus casei* (12)*, Lactobacillus mesenteroides* (13), and *Oenococcus onei* (14), and only one multi-strain reconstruction for *L. reuteri* covering for 36 strains (15). Our work aims to build upon these foundational studies.

In this study, 2,446 GEMs were generated across the *Lactobacillaceae* family. This set of models (called a PanGEM) enables the identification of conserved and variable metabolic traits across different family members. The individual GEMs can be used to develop strategies for strain improvement and bioprocess optimization. Moreover, predictive GEMs have a wide range of applications, from understanding the metabolic basis for probiotic properties to producing value-added compounds for the food and pharmaceutical industries. Overall, constructing the PanGEM is important to fully harness *Lactobacillaceae*’s biotechnological potential, and it constitutes a new quantitative representation of the genomic basis for this large industry.

## RESULTS

### Metabolic network reconstruction and genome-scale models for a family of bacteria

**Fig 1:**
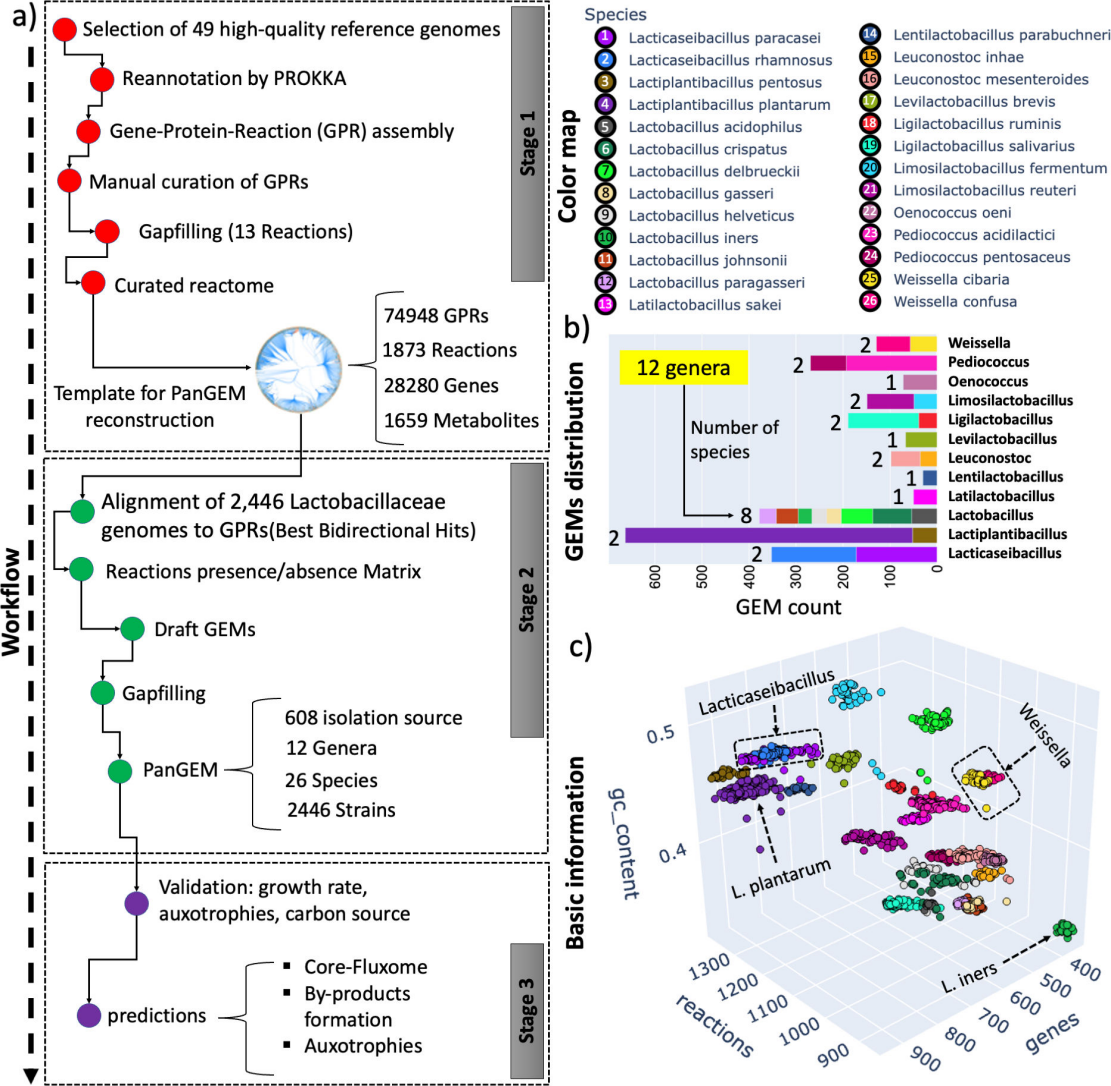
Pangenome-scale metabolic reconstructions for *Lactobacillaceae*. (**a**) The overall three-stage workflow used in this study. Stage 1: reactome reconstruction. To formulate a draft pan-metabolic network reconstruction, 49 *Lactobacillaceae* reference genomes were selected and annotated by Prokka. The draft reactome was manually curated to develop “gold-standard” multi-allelic gene-protein-reaction associations, covering 1,832 reactions and 28,280 metabolic alleles. Stage 2: building strain-specific metabolic reconstructions on a family-wide basis. A total of 2,446 *Lactobacillaceae* genomes from NCBI were aligned with BLAST against the GPRs to identify the corresponding reactions based on sequence similarity. A biomass objective function was added to all draft reconstructions to identify and fill gaps in the metabolic reconstruction. At this step, all GEMs were able to produce biomass and were ready for further analysis. Stage 3: validation and analysis of strain-specific GEMs. Additional validation through iterative refinement was performed to achieve precise and accurate metabolic phenotype predictions. After GEMs passed quality control, analysis was performed to identify common metabolic capabilities and differences across individual *Lactobacillaceae* strains. (**b**) GEM distribution across the *Lactobacillaceae*. GEMs are classified based on their genus (*Y*-axis). Different species within each genus are annotated according to the color map; bar plots are annotated with the number of species within each genus. PanGEM contains 12 genera, including *Lactobacillus*, *Ligilactobacillus*, *Limosilactobacillus*, *Lacticaseibacillus*, *Oenococcus*, *Leuconostoc*, *Levilactobacillus*, *Pediococcus*, *Lactiplantibacillus*, *Latilactobacillus*, *Weissella*, and *Lentilactobacillus*. (**c**) The basic information about the metabolic reconstructions in PanGEM. Color map annotates species, and the three axes depict the number of reactions, the number of genes for each reconstruction, and the GC content of the corresponding genome. These three parameters give distinct clusters for the stains of each species.

First, a metabolic pan-reactome was constructed for use as a template for species-specific metabolic reconstructions. The pan-reactome was reconstructed from 49 high-quality reference genomes, representing 33 species from 9 genera (File S1). The reactome (available at https://github.com/omidard/LactoPanGEM/blob/main/LBReactome.xml) contained 75,299 gene-protein-reaction associations (GPRs), 1,873 reactions (File S2), 28,280 genes, and 1,659 metabolites (File S3) (Fig. 1a, stage 1). Reactions were distributed across 231 cellular subsystems. Of 1,873 reactions, 1,516 were gene-associated reactions, whereas the remaining belonged to other types, including exchange, orphan, gap (see Fig. S1 for a detailed gap analysis), demand, and sink reactions. For clarity, it is worth noting that the 75,299 gene-protein-reaction associations result from capturing all possible alleles for a reaction across our reference genomes. Consequently, multiple distinct gene sets, representing different alleles, can be associated with a single metabolic reaction, leading to several GPRs for that specific metabolic reaction.

Second, metabolic models for the *Lactobacillaceae* family were constructed. The GPRs were matched against the ORFs in every qualified genome, resulting in 2,446 strain-specific GEMs from 26 species and 12 genera obtained from 608 distinct isolation sources (6) (Fig. S2). *L. plantarum* had the highest number of GEMs (611), while *Lactobacillus iners* and *Lactobacillus parabuchneri* had the lowest (30 each). The genus *Lactiplantibacillus* had the most GEMs (663), while *Lentilactobacillus* had the least (30) (see Fig. 1b). In the *Lactobacillaceae* PanGEM, the genus *Lactobacillus* had the highest diversity with eight species, while *Oenococcus, Levilactobacillus*, *Lentilactobacillus*, and *Latilactobacillus* had only one species each, resulting in the lowest diversity within the group.

The PanGEM covers a wide range of metabolic diversity across 26 *Lactobacillaceae* species, with multiple GEMs for each species. The number of reactions per genome ranged from 859 to 1,358, representing 354–944 genes, respectively. On average, *L. iners* had the lowest number of genes and reactions, while *Lactobacillus pentosus* had the highest (see Fig. S3). An in-depth comparison of reaction presence relative to genome length within the *Lactobacillaceae* family is presented in Fig. S4. The analysis reveals a strong correlation (*R*² = 0.87) between genome size and metabolic reactions. Notably, strains with smaller genomes predominantly exhibit lost reactions in lipid, aminoacid, and nucleotide metabolism. Each species can be distinguished from others based on three basic properties (number of genes, number of reactions, and GC content of genomes) (Fig. 1c).

### Validation of PanGEM

**Fig 2:**
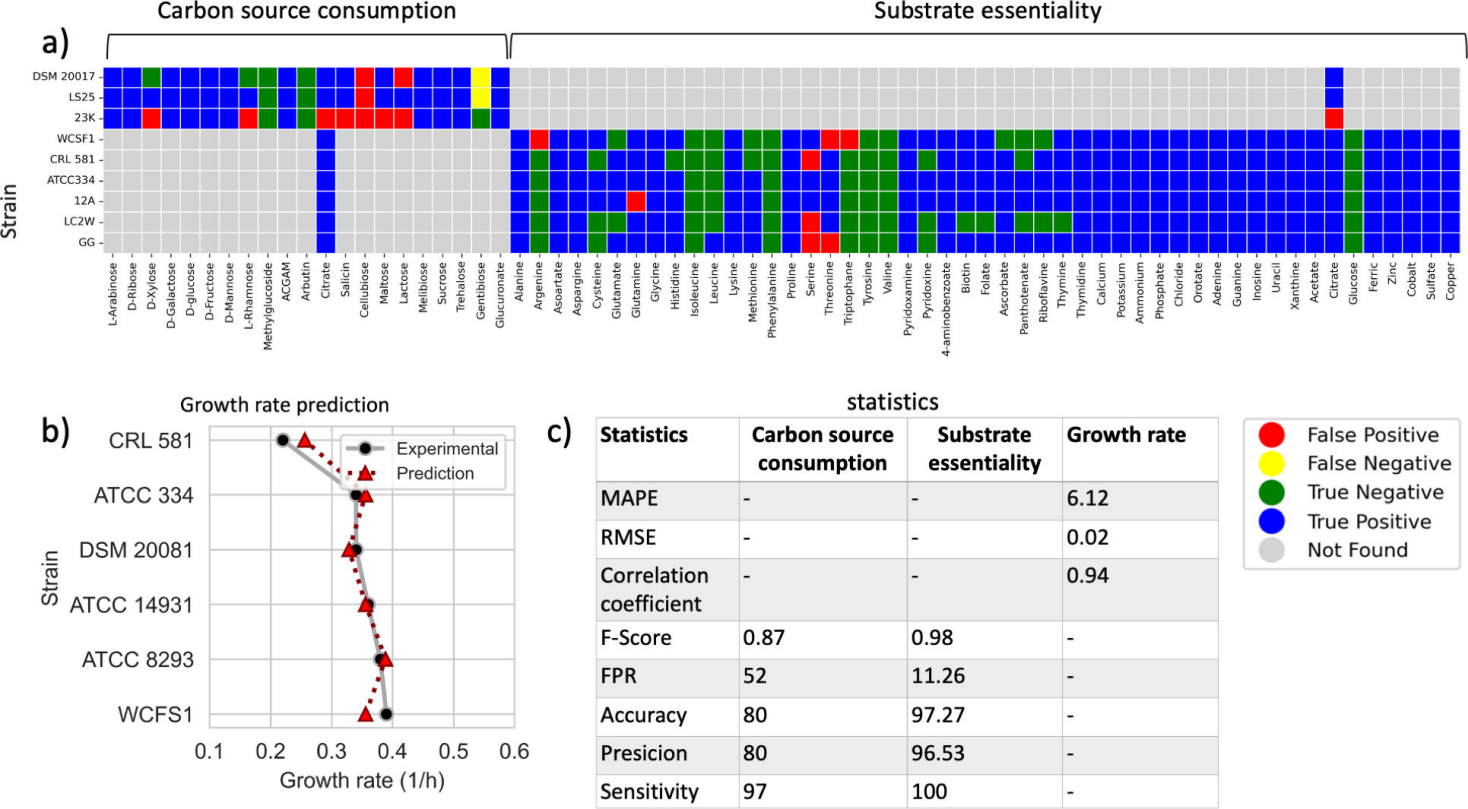
PanGEM validation. (**a**) Carbon source utilization: carbon source utilization validation for three strains within PanGEM (*L. sakei* 23K, *L. sakei* DSM 20017, and *L. sakei* LS25) by comparing experimental and predicted results. The capability to grow on 21 carbon sources (*X*-axis) for three strains (*Y*-axis) is shown; the color map annotates false/true/positive/negative predictions. Statistical analysis based on a confusion matrix showed an *F*-score of 0.98, a false-positive rate (FPR) of 11.26%, a precision of 96.53%, an accuracy of 97.27%, and a sensitivity of 100%. Substrate essentiality: prediction of auxotrophies. Validation of PanGEM by comparing experimental and predicted results for CDM component essentiality for six strains [*L. plantarum* WCSF1, *Lactobacillus delbrueckii* CRL581, *L. paracasei* ATCC334, *L. paracasei* LC2W, and *Lacticaseibacillus rhamnosus* GG (*Y*-axis)]. A single-component omission analysis of 49 components of *Lactobacillaceae*-specific CDM was simulated using flux-balance analysis (FBA), and results were compared with experimental data. Orange dots represent false-positive predictions. Blue and green dots represent true negative and true positive, respectively. No false-negative prediction was observed, with an *F*-score of 0.87, a false positive rate of 52%, a precision of 80%, an accuracy of 80%, and a sensitivity of 97%. (**b**) A comparison of simulated growth rate using FBA on CDM (dashed line-red triangle) with experimental data on the same condition (solid line-black dots) for six strains within PanGEM. CRL 581, *L. delbrueckii* CRL 581; ATCC 8293, *L. mesenteroides* ATCC 8293; DSM 20017, *L. sakei* DSM 20017; DSM 20081, *L. sakei* DSM 20081; LS25, *L. sakei* LS25; 23K, *L. sakei* 23K; WCFS1, *L. plantarum* WCFS1; ATCC334, *L. paracasei* ATCC334; 12A, *L. paracasei* 12A; LC2W, *L. paracasei* LC2W; GG, *Lacticaseibacillus rhamnosus* GG; ATCC 14931, *Limosilactobacillus fermentum* ATCC 14931). (**c**) Statistics. Statistical analysis of GEM validation results is summarized in the table. For quantitative predictions (growth rates), mean absolute percentage error, root mean squared error, and correlation coefficient were calculated. For qualitative predictions (auxotrophy and C-source utilization), *F*-score, FPR, precision, accuracy, and sensitivity were calculated.

Once formulated, GEMs were validated against experimentally determined metabolic and physiological traits (16). Growth predictions on 21 carbon sources for three strains of *Limosilactibacillus* (*sakei* DSM 20017, *sakei* LS25, and *sakei* 23k) were compared to experimental results available in the literature (17) (Fig. 2a). Out of 63 simulations, 52 (83%) were accurate, while 2 were false negatives, and 9 were false positives. Both predictions and experimental results were obtained on chemically defined media (CDM). (see CDM formulation and constraints in Table S2).

For further qualitative phenotypic validation of the PanGEM, single omission analysis of 49 compounds in a CDM was simulated by flux-balance analysis (FBA). The computational results were compared to experimental data (13, 18–21) (Fig. 2a). Experimental data were available for six strains within PanGEM, representing four different species and three unique genera. FBA was performed to predict the single omission of 49 compounds from CDM, one by one across six strains. Among 294 simulations, only 8 (2.7%) failed to predict the correct phenotype (false positives) (Fig. 2a).

Growth rate computations showed that the models were capable of quantitative prediction of growth rates that were in agreement with the experimental reports (19, 22–24) (Fig. 2b). Despite the limited growth rate data available in the literature from CDM, validation was performed on six GEMs, and predictions were satisfactory with a mean absolute percentage error of 6.62, root mean squared error of 0.02, and correlation coefficient of 0.93. These metrics illustrate the prediction potential of PanGEM models (Fig. 2b). The sensitivity of predicted growth rates to variations in growth-associated maintenance (GAM), non-growth-associated maintenance (NGAM), and amino acid uptake rates was rigorously examined. While changes in GAM and NGAM parameters did not show an influence on growth rates (Fig. S5), alterations in amino acid uptake rates were shown to be impactful (Fig. S6).

PanGEM could thus be validated against data reported in the literature (Fig. 2c). The validated PanGEM can be used for the analysis of more detailed metabolic traits.

### Classifying strain-specific reactomes in PanGEM

**Fig 3:**
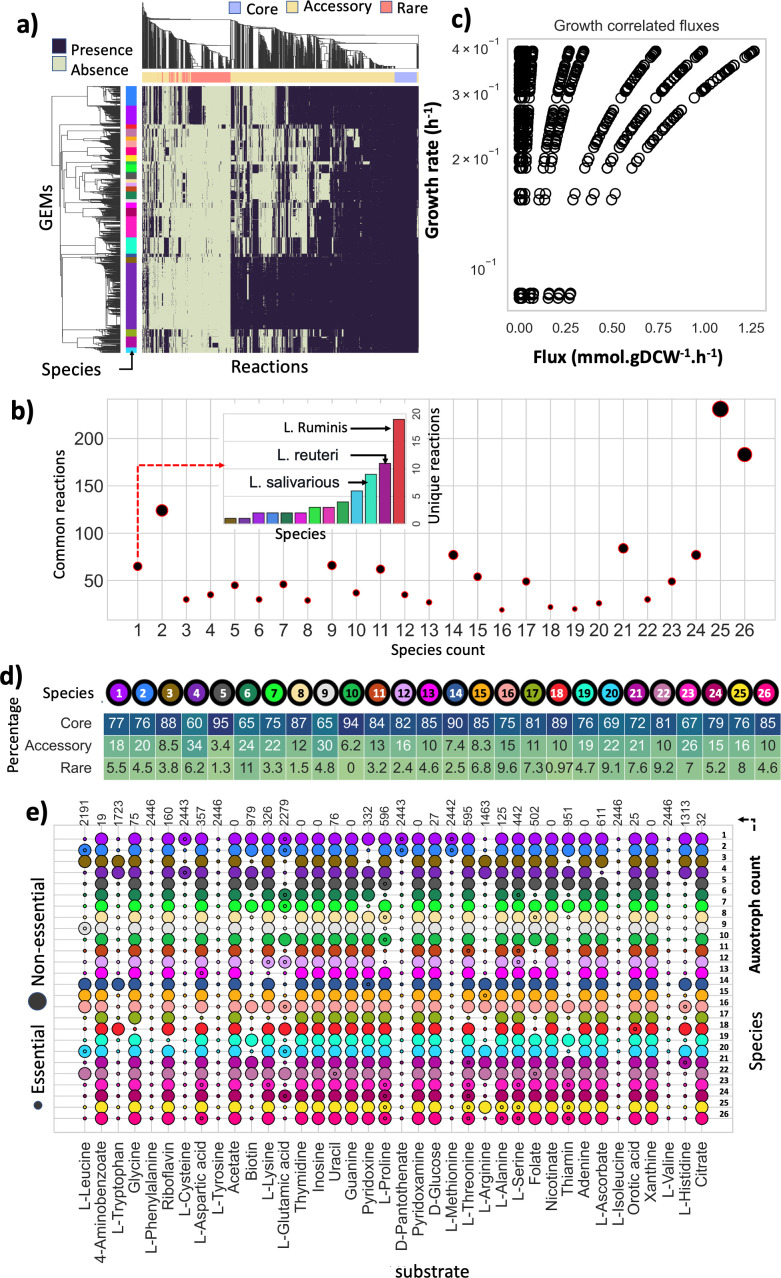
Characteristics of the *Lactobacillaceae* reactome. (**a**) Reaction presence-absence cluster map shows reaction presence and absence calls in each strain (represented by a row) in each species (color-coded as a group of rows). Strain-specific metabolic network reconstructions and reactions are represented by rows and columns, respectively. Row color refers to species; column color represents core, accessory, and rare reactomes. (**b**) Shared reactions’ distribution across the 26 species. Scatter plot depicts the number of common reactions among different species., The inset bar chart represents the unique reaction count per species. Bars are annotated based on the species color map (see species color map in Fig. 1). (**c**) The plot visualizes the growth-correlated fluxes of the core reactome, showcasing the maximum flux of 74 reactions with a correlation coefficient of 1 against the growth rate. Each dot on the plot represents a reaction, with the *x*-axis denoting the maximum flux of reactions and the *y*-axis indicating the growth rates. (d) Intra-species reactions frequency analysis. Heatmap depicts species-specific core, accessory, and rare reactomes, color dot represents species (Fig. 1, species color map), and values within the heatmap are the percentage of intra-species core, accessory, and rare reactomes. (**e**) PanGEM prediction of species auxotrophies in CDM. The figure represents the essentiality of compounds across different strains within a species. Small dots indicate essential compounds, while large dots denote non-essential compounds. When dots are superimposed, it signifies that the compound’s essentiality varies among the strains being essential for some but not all. The color of each dot corresponds to a specific species, as detailed in the color map provided in Fig. 1.

Strain-specific GEMs can be clustered based on the reactions they contain (Fig. 3a). Such clustering highlights metabolic differences between species. The cluster map (Fig. 3a) shows three clusters of reactions: core reactions (common to all strains), accessory reactions (found in many strains), and rare reactions (found in few or even in single strains, then called unique). All strains shared 185 common reactions (Fig. 3b), forming the *Lactobacillaceae* core reactome. There were 1,130 accessory reactions, and they are differentially distributed across the 26 strains (Fig. 3a). Metabolic conservation in each species was assessed by performing a reaction frequency analysis (Fig. 3d). Results indicate that *L. plantarum* had the most metabolically diverse strains, while *Lactobacillus acidophilus* was the most metabolically conserved species (Note S1).

A total of 65 unique reactions were found within 13 species (Fig. 3b, inset bar plot). Among all species, *Lactobacillus ruminis*, a niche-specialized species, had the highest unique reaction count of 19. Next was *L. reuteri* with 11 unique reactions, followed by *Ligilactobacillus salivarius,* with 9 unique reactions (Fig. S7).

### Characterization of allowable flux states using PanGEM

FBA analysis was performed for all 2,446 strain models in PanGEM to better understand *Lactobacillaceae* core metabolism. A subset of 185 reactions spanning 30 distinct metabolic subsystems, common to all GEMs, was identified as the core reactome. Flux variability analysis (FVA) confirmed that all reactions within this subset were active across all strains (Fig. S8), leading to their designation as the *Lactobacillaceae* core fluxome (see File S4). Among these reactions, 74 exhibited a correlation coefficient of 1 with respect to the growth rates of the strains, highlighting their fundamental significance as metabolic signatures within the *Lactobacillaceae*.

Among the 30 subsystems of the core fluxome, arginine biosynthesis, cysteine and methionine metabolism, fatty acid biosynthesis, and purine metabolism have the highest fluxes. Other subsystems within the core fluxome were mostly involved in biomass production, such as pyrimidine metabolism, peptidoglycan biosynthesis, aminosugar metabolism, and glycerophospholipid biosynthesis. Nicotinate and nicotine metabolism and pantothenate and CoA biosynthesis were the only subsystems related to cofactor biosynthesis within the core fluxome.

FBA-predicted CDM media component essentiality for each GEM revealed metabolic similarities and differences in *Lactobacillaceae* (Fig. 3e). *L. plantarum* had the lowest auxotrophy count. *Pediococcus acidilactici* and *Weissella cibaria* had the highest number; *L. sakei* and *Lactobacillus paragasseri* had the most consistent auxotrophies. Isoleucine, valine, phenylalanine, and tyrosine were globally essential in all 2,446 models in PanGEM (Note S2).

The PanGEM enabled the assessment of the composition of the reactome and the range of allowable flux states. The most interesting finding about the reactome composition is the prevalence of species-specific unique reactions. In terms of the core fluxome, reactions exhibited consistent flux across the PanGEM, while certain reactions displayed varying flux values within each individual GEM when compared to the others, such as PPA.

### Exploration of niche adaptation using PanGEM

**Fig 4:**
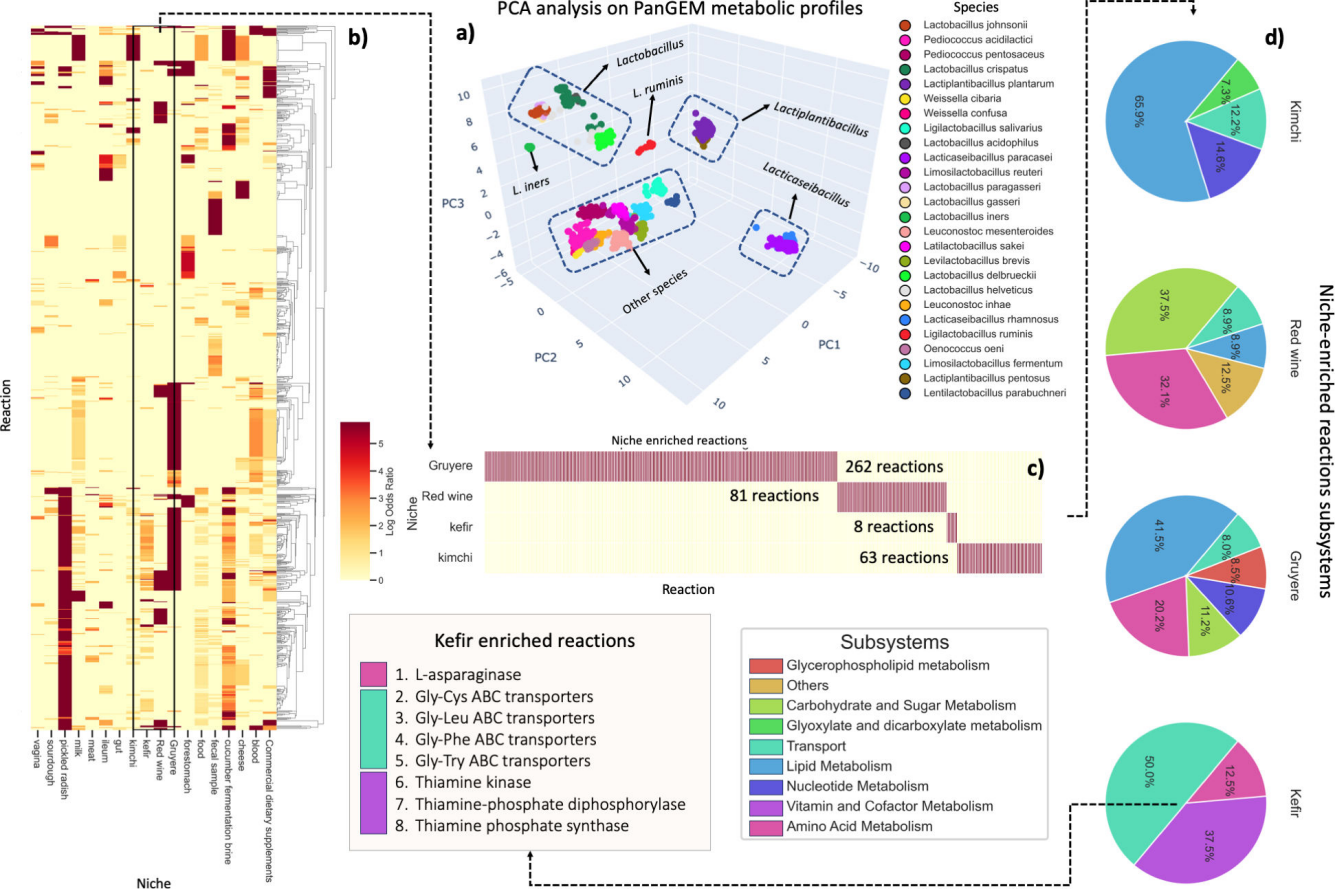
Niche-enriched reactions. (**a**) Three-dimensional PCA plot of 2,446 bacterial strains based on their metabolic reactions, color-coded by species. The plot reveals six distinct clusters, including *Lactobacillus*, *Lactoplantibacillus*, and *Lactocaseibacillus* genus, as well as a separate cluster for *Lactobacillus ruminis* and *Lactobacillus iners*, and a final cluster containing all remaining species. (**b**) Cluster map illustrating niche-enriched reactions specific to isolation sources with more than 10 strains. The color map displays the log odds ratio of reaction prevalence in each niche. (**c**) Focused cluster map on four niches: kefir, red wine, kimchi, and Gruyere. Only reactions with a log odds ratio above five and exclusive to one niche are shown. (**d**) The pie charts depict the reaction distribution in kimchi, kefir, red wine, and Gruyere. Each color represents a distinct cellular subsystem, as indicated in the color map. Below, a list details kefir-enriched reactions.

PCA analysis indicated distinct metabolic patterns across the *Lactobacillaceae* species, as evidenced by the clustering patterns shown in Fig. 4a. These patterns potentially mirror phylogenetic relationships and adaptations to various ecological niches. For instance, while genus-based clustering suggests phylogenetic similarities, species such *as L. iners* and *L. ruminis* diverge from other members of their genus, hinting at unique metabolic profiles.

*Lactobacillus* species are predominantly found in the gastrointestinal tracts of humans and animals. In contrast, *Lactiplantibacillus* species are associated with plant environments like soils and fermenting vegetables. *Lacticaseibacillus* species, on the other hand, are common in dairy settings. Interestingly, *Lactobacillus ruminis* emerged distinctively in the analysis, possibly indicative of specific adaptations within the ruminant digestive systems. Further investigations are essential to solidify these preliminary observations.

Clustering offers insights into a possible connection between bacterial metabolic profiles and their ecological backdrops. A deeper exploration of these associations could shed light on the unique roles these bacteria undertake in diverse habitats.

To quantitatively assess the relationship between metabolic profiles and their respective niches, we conducted a PERMANOVA analysis using reaction content Jaccard distances as the response variable. The PERMANOVA revealed significant effects of both isolation source and genus on reaction content variation. Isolation source explained 26.5% (*R*^2^ value of 0.256) of the total variation (*P* < 0.001), while genus (*R*^2^ value of 0.912) explained 91.2% (*P* < 0.001). Despite the stronger effect observed for the genus, the isolation source remained statistically significant. Further analyses were performed to identify niche-enriched reactions from sources with more than 10 strains (detailed in Fig. 4b and File S5).

For illustrative purposes, four niches were examined: kefir, red wine, kimchi, and Gruyere (Fig. 4c). It was observed that most reactions enriched in kimchi and Gruyere are associated with lipid metabolism. In red wine, reactions related to carbohydrate and sugar metabolism were predominantly enriched, while in kefir, an enrichment of transporters was noted (Fig. 4d).

Upon closer examination of kefir-enriched reactions, eight were identified. Of these, four were found to be involved in the transport of dipeptides. These dipeptides have been shown to be produced by yeast and consumed by *Lactobacillaceae* during kefir fermentation (25). Three of the reactions have been associated with thiamine metabolism, in line with the high vitamin B1 content that has been reported in kefir (26). Furthermore, the enrichment of asparaginase in kefir can be correlated with the depletion of asparagine that has been observed during its fermentation (27).

### Biotechnological potential of *Lactobacillaceae* can be discovered using PanGEM

**Fig 5:**
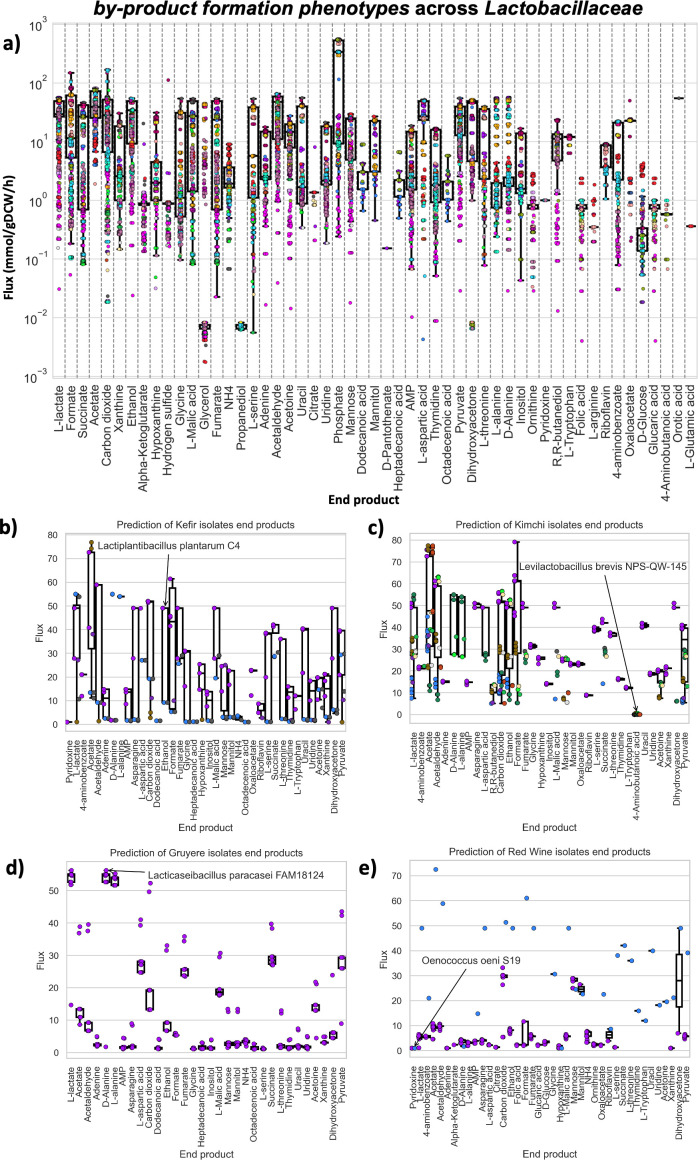
PanGEM prediction of by-product formation phenotypes. (**a**) Prediction of maximum and minimum by-product secretion rates across PanGEM models. The box plot illustrates the capability of different species for product formation, with species represented by color-coded dots. (**b**) By-product profiling of kefir isolates. (**c**) By-product profiling of kimchi isolates. (**d**) By-product profiling of Gruyere isolates. (**e**) By-product profiling of red wine isolates. Across all figures in this panel, dots symbolize species, color-coded according to the species color map shown in Fig. 1. The *Y*-axis depicts flux ranges, calculated using FVA on CDM. The *X*-axis displays the secreted metabolites. Key by-products specific to the source of isolation are highlighted, with annotations indicating their predicted optimal producers.

Validated and characterized PanGEM models can be used for a variety of applications (28, 29). Metabolic by-product secretion is a defining metabolic trait of *Lactobacillaceae* and determines their uses in food production. FVA was performed to predict potential metabolite production across *Lactobacillaceae* (Fig. 5). As expected, PanGEM models showed high production rates for lactate and acetate (Fig. 5a). Metabolite connectivity analysis across all metabolic networks of PanGEM showed that the diversity of *Lactobacillaceae* end-products had a strong correlation with the connectivity of pyruvate and glutamate (Fig. S9c).

PanGEM predicted the production of several flavor compounds, such as acetoin, acetaldehyde, pyruvate, succinate, D-alanine, and ethanol, as well as the neurotransmitter gamma-aminobutyric acid (GABA). *L. paracasei* strains were predicted to be major D-Alanine producers. Ethanol producers were mostly plant-based *L. pentosus* and *Lactobacillus brevis*, dairy-based *P. acidilactici,* and commensal *L. paracasei*. Succinate producers include commensal *Lacticaseibacillus rhamnosus, P. acidilactici,* and *L. paracasei* isolated from unknown sources. GABA production was predicted in two species, commensal *P. acidilactici* and commensal *L. brevis* (Fig. S7a). PanGEM thus predicts the metabolic by-product formation on a species-specific basis.

To understand the biotechnological capabilities of product-specific isolates and how they contribute to desirable organoleptic properties of the final product, FVA was performed on GEMs belonging to isolates from four industrial products, including kimchi, kefir, Gruyere, and red wine. FVA predicted different fermentation profiles for each product. The kefir niche features two types of kefir: Tibetan kefir and a category generally labeled as “Kefir.” Within this niche, a total of 15 isolates have been identified. The isolates include nine strains of *L. plantarum*, two of *L. paracasei*, two of *L. rhamnosus*, one of *L. mesenteroides*, and one of *Pediococcus pentosaceus*. These strains collectively have the capability to produce 40 different metabolites. Among these, ethanol, succinate, acetoin, and lactic acid have been noted as important metabolites due to their roles in kefir’s nutritional profile and fermentation process (Fig. 5b).

The red wine niche includes a selection of four wine types: Chinese red wine, Nero di Troia red wine, Patagonian red wine, and a category broadly labeled as red wine. Within this selection, there are 22 strains, with 21 identified as *O. oeni* and one as *L. plantarum*. These strains are notable for their broad range of metabolite production. Specifically, among the 40 potentially producible metabolites, pyridoxine, and ornithine stand out for their importance in the wine industry, as illustrated in Fig. 5e.

The kimchi niche includes a collection of 14 unique types, including varieties such as napa cabbage kimchi and white kimchi (baek kimchi). PanGEM includes 74 strains from 10 different species within this profile. The species include *L. plantarum* (34 isolates), *W. cibaria* (12), *L. sakei* (6), *L. paracasei* (6), *L. mesenteroides* (6), *L. brevis* (4), *P. acidilactici* (2), *Weissella confusa* (1), *L. rhamnosus* (1), and *Limosilactobacillus fermentum* (1). These strains are potentially capable of producing 50 metabolites as predicted by FVA, with formate, acetaldehyde, acetoin, succinate, and GABA being particularly noteworthy for their roles in flavor development and potential health benefits in kimchi (Fig. 5c).

In the specialized niche of Gruyere cheese, we have identified a single, crucial bacterial species, *L. paracasei*, represented by 12 isolates. These isolates are responsible for the production of 50 different metabolites. Notably, formate, acetoin, and succinate have been highlighted as important metabolites, contributing significantly to the characteristic flavor and texture that define Gruyère cheese. Also, interestingly, a high production rate for D-alanine was predicted. Since Gruyere is known for its sweet taste (30) and D-alanine is a known amino acid-based sweetener (31), there might be a link between this phenotype and Gruyere’s organoleptic properties (Fig. 5d).

The pangenome metabolic reconstructions and the metabolic traits that they predict for 2,446 strains were thus highly consistent with the characteristics of the genera and species to which they belong. PanGEM thus provides a global atlas of the genetic basis for metabolic traits in the *Lactobacillaceae* family. One can select an isolation source, identify strains specifically from that source, and conduct FVA to construct an end-product profile. This profile can then be a valuable guide for directing subsequent experimental assessments.

## DISCUSSION

*Lactobacillaceae* are widely used in pharmaceutical, fermentation, and beverage industries (Table S1). As producers of various compounds, such as lactic acid, acetic acid, succinic acid, diacetyl, acetoin, GABA, sorbitol, mannitol, butanediol, propanediol, and the vitamin B family, lactic acid bacteria have a strong potential for biotransformation. Despite their enormous industrial potential, prominent market share, and the availability of large amounts of omics data (32–35), we lack computational models that link genotypes to desirable industrial phenotypes. This study remedies this shortcoming by using pangenome analysis and metabolic reconstruction to characterize the metabolic potential of the *Lactobacillaceae* by generating 2,446 high-quality GEMs across 26 species obtained from 608 isolation sources.

Reactome analysis demonstrated that the core reactome comprises only 8% of the total reactome. In comparison, the accessory and rare reactomes account for 73% and 19%, respectively, indicating significant diversity in metabolic capabilities among *Lactobacillaceae*. Of the 65 unique reactions identified in the reactome, *L. ruminis, L. reuteri*, and *L. salivarius* have the highest number. These species commonly reside in the human gut and oral cavity, suggesting that acquiring novel metabolic capabilities may be crucial for bacterial adaptation and survival in complex environments. *Lactobacillus ruminis* demonstrates the highest count of unique reactions, comprising a total of 19. Certain unique reactions, such as TEICEXP, TEICEXP2, and TEICEXP3 related to teichoic acid export, were only found in *Pediococcus acidilactici*, which may be a distinct trait of this species. Previous studies have indicated that the thick cell wall of *P. acidilactici* plays a crucial role in heavy metal accumulation in the gut (36). *P. acidilactici and L. brevis* were also responsible for approximately 70% of microbial spoilage in beer due to hop resistance linked to the cell wall and teichoic acid structure (37). Additionally, choline trimethylamine-lyase was found only in *L. plantarum* strains. This enzyme cleaves choline to produce trimethylamine (TMA) and acetaldehyde. TMA causes several disease-associated microbial metabolites (38). Therefore*,* PanGEM could screen for disease-associated microbial metabolites such as TMA and their producers to avoid or reduce their usage in food industries.

Analysis of the core fluxome shows the possible activity states of core metabolism. Its properties revealed a consistent and narrow flux range (−0.15–1.26 mmol/gDCW/h) for a subset of 74 core reactions, which is predicted to have a maximal correlation with growth rate across *Lactobacillaceae*. However, 111 reactions showed high flux variation (−1,000–1,000) across different strains indicating a flexible part of the core reactome (Fig. S8b).

FBA predicted global auxotrophy, including isoleucine, valine, phenylalanine, and tyrosine, indicating a family-wide lack of complete biosynthetic pathways for these amino acids. This information could be used to generate strain-specific minimal media and screen for wild-type auxotroph strains as a predictive tool to prevent the backslopping of industrially important strains.

FVA was also performed to screen for the biotechnological potential of the species in PanGEM. As expected, PanGEM showed high production rates for lactate and acetate, which are well-known as primary organic acids produced by lactic acid bacteria (39). PanGEM predicted the production of 54 compounds across all strains, some of which are biotechnologically important compounds, consistent with the reports in the literature. These include acetoin (40), acetaldehyde (41), pyruvate (42), succinate (43), D-alanine (44), mannitol (45), formate (46), malate, citrate (46), propanediol (47), butanediol (48), ethanol (49), as well as the neurotransmitter GABA (50), and vitamins such as riboflavin (51) and pyridoxal phosphate (52).

PanGEM’s analysis provides insight into niche-specific adaptations among *Lactobacillaceae* species. A targeted examination of enriched reactions in niches like kimchi, Gruyere, red wine, and kefir highlighted distinct metabolic pathways: lipid metabolism in kimchi and Gruyere, carbohydrate processes in red wine, and transport mechanisms in kefir. Notably, within kefir, reactions related to dipeptide transport and thiamine metabolism align with observed fermentation properties and vitamin B1 content. This framework underscores PanGEM’s value in identifying reactions that potentially enhance bacterial fitness in specific environments.

Using PanGEM, we investigated the biotechnological capabilities of product-specific isolates and their link to the organoleptic properties of the final product. GEMs for kimchi, kefir, Gruyere, and red wine isolates were analyzed, and PanGEM predicted the fermentation profile of related strains to identify their role in the quality of the final product. PanGEM contained 74 kimchi isolates belonging to 10 different species predicted to be capable of producing 50 different metabolites, including succinate responsible for umami taste (53), acetic acid as the main organic acid (54), acetaldehyde (55), and acetoin (56). Moreover, PanGEM predicted GABA production, which is one of the targeted metabolites for overproduction during kimchi fermentation (57). Also, red wine isolates, consisting of 21 *Oenococcus onei* strains and 1 *L. plantarum strain*, were predicted as producers of malate (58), pyridoxine (59), and ornithine (60, 61). The kefir isolates comprised 15 strains from five species, with FVA predicting the production of 40 metabolites, including ethanol (62), succinate (63), acetoin (64), and lactic acid (64). Gruyere isolates all belonged to *L. paracasei*, predicted to be capable of the production of 50 different metabolites, including acetoin (65) and succinate (66). Interestingly, a high production rate for D-alanine was predicted, which could be related to the sweet taste of Gruyere (31).

Although PanGEM shows a high degree of precision in predicting the metabolic traits of *Lactobacillaceae*, transporter annotation remains a challenging aspect in genome-scale metabolic model reconstructions. The accuracy of such annotations directly impacts the prediction of carbon source utilization. While our model demonstrates commendable precision in amino acid essentiality predictions, our findings in carbon source utilization highlight the need for enhanced attention to transporter annotation for some strains. Achieving optimal precision and accuracy will require continued refinement and perhaps even new methodologies to improve transporter annotations. Such improvements are vital for the accurate prediction of metabolic capabilities and will play a key role in future iterations and refinements of GEMs. A fundamental reason for PanGEM’s predictive power is the relative simplicity of *Lactobacillaceae* metabolism and that the GPRs reflect its genomic basis well. Another challenge in pangenome-scale reconstructions of metabolism is the absence of comprehensive experimental data regarding biomass composition for every species under study. This limitation necessitates the generalization of biomass objective functions (BOFs), which can introduce varying degrees of predictive inaccuracies in model simulations, directly correlated to the degree of BOF variability among different strains. The development of species-specific BOFs, as opposed to the application of generalized ones, could significantly enhance the precision of GEMs. Despite this potential improvement, the dearth of detailed biomass composition data persists as a fundamental constraint within the field, which could be addressed in future studies. Despite this limitation, PanGEM provides a structured and computable genetic basis for metabolic traits in the *Lactobacillaceae* family.

The economic impact of *Lactobacillaceae* is enormous, enabling industries with an annual turnover exceeding a trillion U.S. dollars. Our study presents a family-wide metabolic reconstruction of *Lactobacillaceae* using pangenome analysis and metabolic reconstruction, resulting in high-quality genome-scale metabolic models. The validated PanGEM enables the discovery and understanding of the biotechnological potential of these bacteria, developing novel applications and screening for disease-associated microbial metabolites in the food industry.

## MATERIALS AND METHODS

### Reactome reconstruction

#### Data collection

For the reconstruction of the *Lactobacillaceae-*specific reactome, 49 reference genomes (see Note S4 for reference genome selection rationale) (File S1) were downloaded from NCBI covering 9 genera and 33 species to capture the metabolic diversity of *Lactobacillaceae* as much as possible. We chose the 49 reference genomes based on two main factors: data availability and the presence of existing genome-scale metabolic models.

#### Genome re-annotation

Initially, all genomes were annotated using the Prokka software (67), and stringent parameters were applied (66). A group of 13 high-quality manually annotated genomes was selected from the NCBI GenBank database as reference genomes for annotation (File S6). The output GenBank files were merged into a single GenBank file and used as the reference genome of the reactome.

#### GPR formulation and curation

Metabolic functions were assigned to the reference genome of the reactome using the Modelseed (68) pipeline to formulate the first draft of reactome. Subsequently, all reactions went through a manual curation process. A cross-validation was performed for each reaction against the KEGG universal reactome. GPR boolean rules were checked against BioCyc (69) and KEGG databases (70). Reactions and metabolite identifiers were mapped against the BIGG database (71). The directionality of each reaction was checked based on (i) Gibbs free energy retrieved from Biocyc, (ii) BIGG reactions due to the availability of information, and (iii) previous *Lactobacillaceae* GEM reconstructions (10, 12, 14, 15, 20, 24). Metabolite charges were curated based on (i) BIGG metabolites, (ii) KEGG metabolites, (iii) ChEBI metabolites, and (iv) previous GEMs. Where information was inconsistent across databases, Marvine’s suite was deployed to calculate metabolite charges at physiological pH (7.2). Reactions mass and charge balance were checked using the Cobrapy package (72) and were curated where necessary. Cellular subsystems were assigned to the reactions based on KEGG subsystems.

#### Reactome refinement

The curated reactome was subjected to an iterative refinement process; for this goal, (i) spontaneous reactions (non-enzymatic reactions) were extracted from KEGG, BIGG, and previous reconstructions and were added to the draft reactome; (ii) exchange reactions were extracted from BIGG and previous reconstructions and were added to the draft reactome; (iii) genes missing from the reactome were identified and assigned to their corresponding reactions manually (Note S5) to form a GPR and included in the draft reactome; (d) a bidirectional blast with a similarity threshold of 60% was performed on the reference genome of reactome against BIGG reconstructions to find and add further missing reactions; (e) a BOF was taken from *L. plantarum* reconstruction (10) as a Universal *Lactobacilli*-BOF; (f) refined reactome was converted to a mathematical model using Cobrapy; (g) a primary gapfilling was performed on the reactome to find and fill metabolic gaps (Fig. S7) within the reactome using fastgapfill algorithm (73). Existence of thermodynamically infeasible cycles (TICs) was checked after curation of reaction directionality in our reactome by conducting a flux variability analysis without any assigned objective function. Having no active fluxes within the network confirmed the absence of TICs in reactome, ensuring the accuracy and reliability of the GEMs derived from this foundation. The output of this step is called *Lactobacillaceae* reactome hereafter and was used as a template for strain-specific GEM reconstruction across *Lactobacillaceae* members. Non-growth-associated maintenance was set to 1 mmol/gDCW/h, this value was calculated based on the mean NGAM of publicly available *Lactobacillaceae* GEMs (Table S3)

### Lacto PanGEM reconstruction

#### Genome collection and re-annotation

*Lactobacillaceae* genomes were downloaded from NCBI. following quality control steps were performed during genome selection (6):

1. A total of 4,783 genomes of the *Lactobacillaceae* family were retrieved from the NCBI database.
2. Genome Taxonomy Database Toolkit was deployed to re-annotate the taxonomy for all 4,783 genomes (74).
3. Furthermore, quality control and quality assurance (QC/QA) were done to get good-quality genomes. The QC/QA includes the taxonomy, number of contigs (<200), and *N*_50_ (>50,000).
4. Species with less than 30 genomes were excluded to maintain sample distribution.
5. Finally, a total number of 2,446 high-quality genomes of the *Lactobacillaceae* family remained to be passed to the multi-strain GEM reconstruction workflow as input (File S7).

Our selection of *Lactobacillaceae* species was based on a specific set of criteria established in our previous study (6). With the continuous growth in the availability of sequenced genomes, some species not included in our current research could be considered for inclusion in future updates of the *Lactobacillaceae* PanGEM.

#### Multi-strain reconstruction

GPR mapping was conducted using bidirectional best hits with a similarity threshold of 70%. For this goal, quality-controlled genomes from the previous step were used as the target genome, and the reference genome of the reactome was used as a reference. The resultant homology matrix was transformed into a GPR presence-absence matrix (74). Mapped GPRs for each strain were collected to generate draft GEMs. Subsequently, all orphan, exchange, and spontaneous reactions, along with the *Lactobacilli*-specific biomass reaction, were added to all drafts.

#### Gapfilling

Gapfilling analysis was performed on generated GEMs to ensure their functionalities. Reactome was used as a collection of candidate reactions for gapfilling; all GEMs were gapfilled on CDM. Initially, GEMs were gapfilled using fastgapfill, although it could not return a possible solution for most GEMs. Subsequently, an alternative methodology (Fig. S8), enabling large-scale gapfilling in a feasible timespan, was developed and applied.

### Lacto PanGEM analysis

#### Dependencies

All analyses were performed in Python language deployed on an Azure virtual machine, statistical analysis was done using Python packages, including pandas, numpy, and scipy, and figures were generated using Matplotlib and Plotly. Cobrapy was used for GEM analysis. In all FBA-based simulations, biomass reaction was defined as an objective function.

#### Niche classification

Isolation sources were collected from NCBI biosample when available, and a total number of 608 distinct isolation sources were identified and classified into nine larger groups including, plant-based, meat-based, dairy-based, commercial, commensal, environmental, beverages, uncategorized (undefined) food, and not reported (Fig. S1).

#### Niche-enriched reactions

We employed the Scikit-learn package to perform principal component analysis (PCA) on our data set, aiming to reduce its dimensionality for visualization purposes. We set the PCA to produce three principal components. Following this, we used the KMeans clustering algorithm from Scikit-learn to identify distinct clusters within the data, setting an optimal cluster count of nine based on the nine major niches. We conducted a chi-square test using the Scipy package to investigate the relationship between metabolic profiles and their respective niches. This statistical test allowed us to quantitatively assess the association between the two variables, providing insights into potential niche-specific metabolic adaptations. We calculated the odds ratio for a more granular understanding of the association between specific metabolic reactions and niches. This was achieved by constructing 2 × 2 contingency tables for each reaction-niche combination and applying Fisher’s exact test.

#### Defining family-wide and species-specific core, accessory, and rare reactomes

A reaction presence-absence matrix was constructed based on the *Lactobacillaceae* reactome and the reaction content of each strain. Family-wide core (*F* > 99%), accessory (15 ≤ *F* ≤ 99), and rare (0 §amp;lt; *F* §amp;lt; 15) reactomes were defined based on reaction frequency (*F*) across PanGEM. To understand the sensitivity of core, accessory, and rare thresholds on the distribution of reactions in reactome categories, we changed the threshold for the core based on the elbow method (98.8%); this did not change the distribution of reactions in reactome categories. Results are included in Fig. S10. Similar parameters were considered for species-specific core, accessory, and rare reactomes. Reactions that were exclusive to a species (whether they could be found in one or multiple strains across a species) were called unique reactions.

### Validation

Validation was carried out over three different data types, including carbon source utilization, auxotrophy, and growth rate.

#### Carbon source utilization

The capability of three strains for growing on 21 different C-sources was predicted and compared to the experimental results obtained from the literature. To simulate growing on different C-sources, the exchange reaction of glucose was bound to zero, and the lower bound of the exchange reaction of the target C-source was bound to 1,000 mmol/gDCW/h. Growth was reported if the predicted growth rate was above 0.01/h. FBA was used for growth simulation.

#### Auxotrophy

Auxotrophy prediction was performed over six strains by bounding the lower bound of each of the 49 CDM components to zero. Auxotrophy was reported for a component if the predicted growth rate was below 0.01/h. FBA was used for growth stimulation.

#### CDM formulation

The lower bound of the glucose exchange reaction was taken from the experimentally measured glucose uptake rate of *L. reuteri* (11). Lower bounds of amino acids’ exchange reaction were set to 1 mmol/gDCW/h except for glutamate and aspartate, which were set to 2 mmol/gDCW/h (see Fig. S11 and Table S2 for a detailed explanation).

#### Growth rate

The growth rate was predicted for five strains using FBA on CDM. Predicted growth rates were compared to the experimental results obtained from the literature.

### Prediction of fermentation profile

Fermentation profile for each strain within PanGEM was predicted by FBA on CDM. Strains were grouped based on isolation sources and species. Strains that were isolated from four industrially important food products were chosen to predict their fermentation profile and find a link between their metabolic capability and the organoleptic properties of their isolation source. A set of metabolites whose exchange reaction was above zero was reported as a fermentation profile for each strain.

## Data Availability

All data and scripts are available at https://github.com/omidard/LactoPanGEM.

## SUPPLEMENTAL MATERIAL

File S1 msystems.00156-24-s0001.xlsx Reactome reference genome data.

File S2 msystems.00156-24-s0002.xlsx Reactome's reactions.

File S3 msystems.00156-24-s0003.xlsx Reactome's metabolite information.

File S4 msystems.00156-24-s0004.xlsx Corefluxome.

File S5 msystems.00156-24-s0005.xlsx Niche-enriched reactions.

File S6 msystems.00156-24-s0006.xlsx Genomes used for re-annotation using PROKKA.

File S7 msystems.00156-24-s0007.xlsx *Lactobacillaceae* genome metadata.

Supplemental information msystems.00156-24-s0008.docx Supplemental figures, tables, and notes.

ASM does not own the copyrights to Supplemental Material that may be linked to, or accessed through, an article. The authors have granted ASM a non-exclusive, world-wide license to publish the Supplemental Material files. Please contact the corresponding author directly for reuse.
