# Supplementary material for: Pangenome reconstruction of Lactobacillaceae metabolism predicts species-specific metabolic traits
Source: mSystems. 2024 Jun 26;9(7):e00156-24. doi: 10.1128/msystems.00156-24 (PMC11265412; doi:10.1128/msystems.00156-24)
Supplement: Supplemental information — Supplemental figures, tables, and notes. [file msystems.00156-24-s0008.docx]

Pangenome reconstruction of *Lactobacillaceae* metabolism predicts species-specific metabolic traits.

**Supplementary Information**

1. Supplementary Figure 1: Gap analysis and gap frequency within Lactobacillaceae PanGEM.
2. Supplementary Figure 2: Scale and diversity of PanGEM for Lactobacillaceae.
3. Supplementary Figure 3: Comparative Analysis of six Genome and GEMs Characteristics Across Lactobacillaceae.
4. Supplementary Figure 4: Detailed Comparative Analysis of Reaction Presence in Relation to Genome Length in Lactobacillaceae.
5. Supplementary Figure 5: Predicted Growth Rate Sensitivity to ATP Maintenance Parameters in GEMs.
6. Supplementary Figure 6. Sensitivity Analysis of Predicted Growth Rates to Amino Acid Uptake Rates on Chemically Defined Media (CDM).
7. Supplementary Figure 7: Lactobacillaceae unique reactions.
8. Supplementary figure 8. Analysis of the Corefluxome in Lactobacillaceae Pan-Genome Models.
9. Supplementary Figure 9: by-product formation prediction.
10. Supplementary Figure 10: Schematic diagram of the gap-filling procedures used for the reconstruction of 2446 GEMs.
11. Supplementary Figure 11: Distribution of essential reactions across metabolic pathways.
12. Supplementary Figure 12: The sensitivity of categorizing reactions as core, accessory, or rare within the reactome relative to the threshold values designated for these classifications.
13. Supplementary Figure 13: Distribution of Amino Acid Uptake Rates as Determined by Flux Variability Analysis (FVA).
14. Supplementary Table 1 calculated the mean NGAM based on published LAB GEM’s NGAM.
15. Supplementary Table. 2. Global market size of Lactobacillaceae-related products.
16. Table. 3. Exchange reaction constraints for CDM simulation by FBA/FVA.
17. Supplementary Note 1. Species-specific reactome.
18. Supplementary Note 2. Predicted essentiality of CDM media components.
19. Supplementary Note 3. Distinct metabolic profiles of Lactobacillaceae.
20. Supplementary Note 4. Reference Genome Selection and Curation Process
21. Supplementary Note 5. Manual mapping of missing reactions.


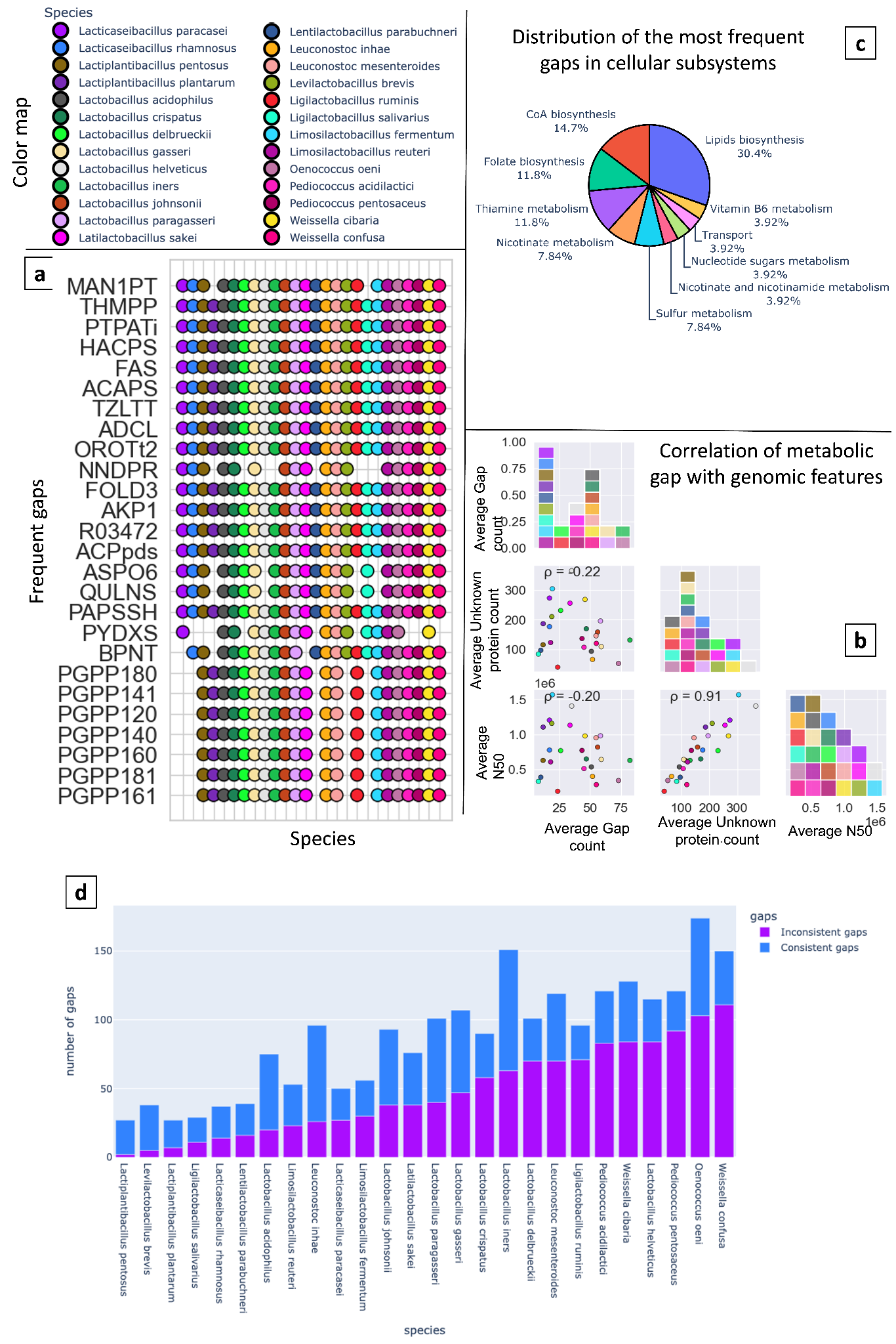


***Supplementary Figure 1: Gap analysis and gap frequency within Lactobacillaceae PanGEM****.* ***a)*** *Most common metabolic gaps within Lactobacillaceae PanGEM, categorized based on gaps frequency in each species. Species are shown on Y-axis, and X-axis shows reactions that have been identified as gaps and have been added to GEMs. Colored dots show the presence of each gap within GEMs, and each color is assigned to a species (color map). b) correlation of gap count with genome contiguity (N50) and knowledge/technical gap (Unknown protein). Colors are coded based on the color map. c) distribution of the most common gaps within cellular subsystems. Values are presented as percentages. d) Stacked bar chart showing consistent and inconsistent gaps in Lactobacillaceae pan-genome models (Lactobacillaceae PanGEM). The y-axis represents the gap count, and the x-axis shows the species analyzed. Inconsistent gaps are present in some, while consistent gaps are present in all strains of a species*


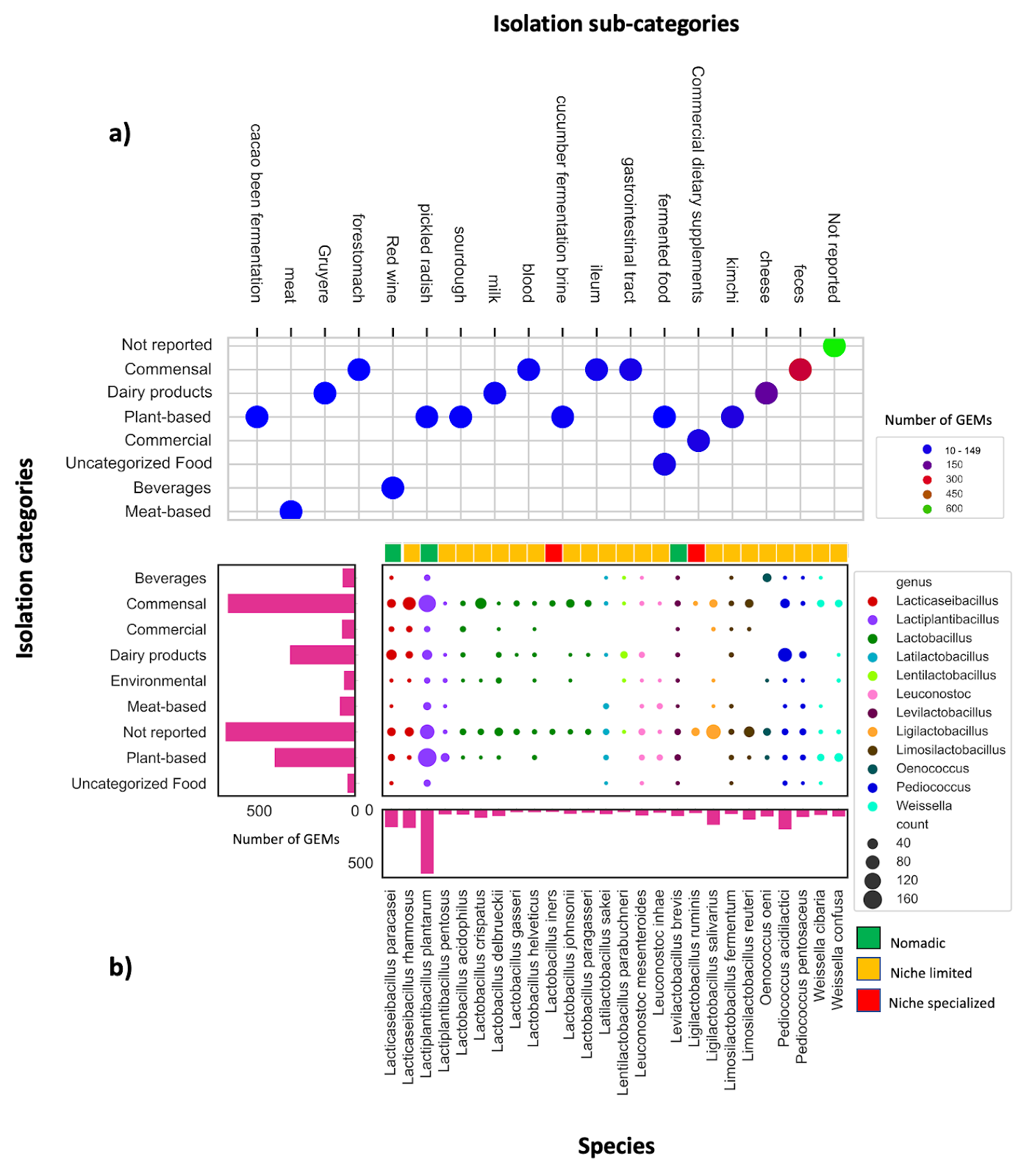


***Supplementary Figure 2: Scale and diversity of PanGEM for Lactobacillaceae****. Panel (a) displays the distribution of GEMs within sub-categories for each major isolation category, revealing significant diversity within the Lactobacillaceae. Panel (b) further highlights the diversity of 2,447 GEMs across nine different isolation sources, 26 species, and multiple genera. The y-axis shows major isolation sites, with colored dots representing GEMs belonging to each genus. The size of the dots corresponds to the number of isolates, while the color bar at the bottom indicates the niche specialization status of each species based on the number of unique isolation categories. The marginal plot on the x-axis shows the number of GEMs within each species, while the marginal plot on the y-axis depicts the number of GEMs within each major isolation source. This figure underscores the vast diversity of Lactobacillaceae and their potential for various applications in industry and biotechnology.*


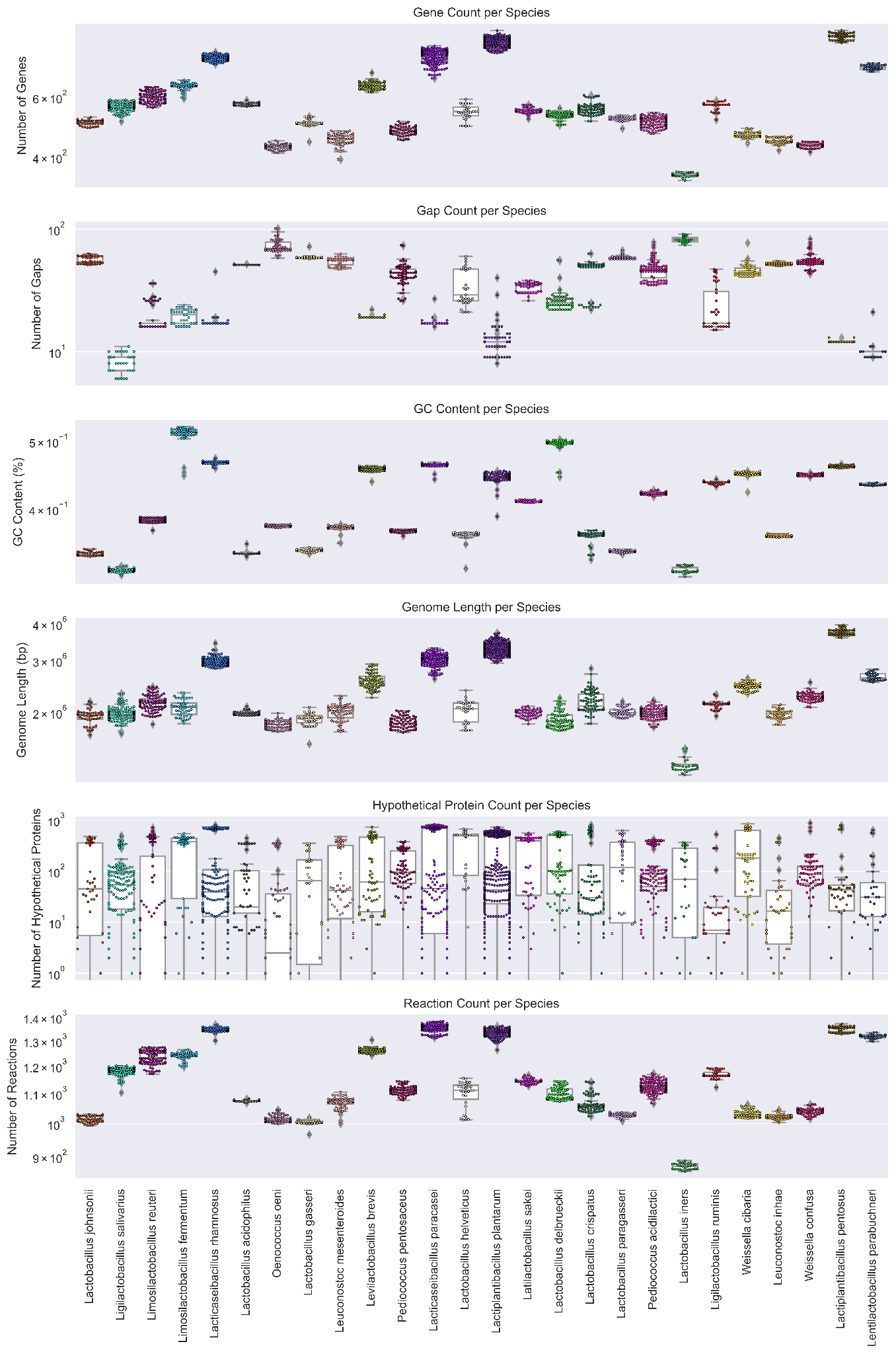


***Supplementary Figure 3: Comparative Analysis of six Genome and GEMs Characteristics Across Lactobacillaceae****. The figure consists of six subplots depicting the distribution of various genome characteristics across multiple bacterial species. The subplots include (A) reaction count, (B) gene count, (C) gap count, (D) GC content, (E) genome length, and (F) number of hypothetical proteins. Each boxplot shows the distribution of the respective GEMs characteristics across the selected bacterial species. The x-axis in each subplot shows the name of the bacterial species, while the y-axis represents the respective characteristic measured.*

*
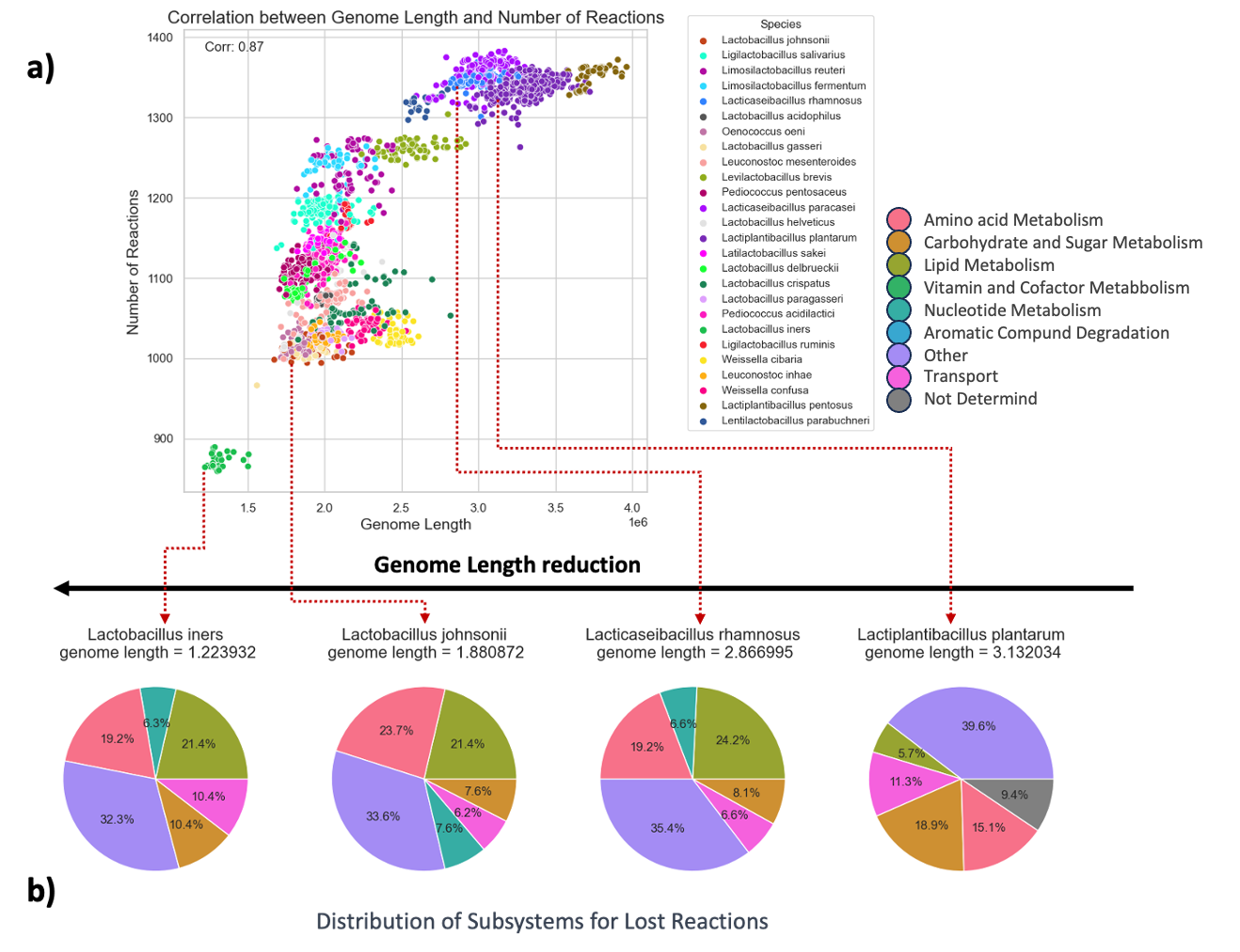
*

***Supplementary Figure 4: Detailed Comparative Analysis of Reaction Presence in Relation to Genome Length in Lactobacillaceae.*** *a) Scatter plot illustrating the relationship between genome length and the number of associated reactions in different genomes of the Lactobacillaceae family. Each data point corresponds to a specific genome, with its size represented on the x-axis and its number of reactions on the y-axis. A linear regression analysis displays a significant correlation coefficient of 0.87, underscoring the importance of genome size in the metabolic diversity and complexity of these bacteria. b) Series of pie charts representing various Lactobacillaceae genomes of differing lengths, showcasing the distribution of reactions that were 'lost' compared to L. pentosus, which has the largest genome size among the studied strains. Each chart is associated with a genome of a particular size, with the segments of the pie chart detailing the metabolic pathways from which reactions are absent. Lipid Metabolism, Amino Acid Metabolism, and Nucleotide Metabolism emerge as the primary pathways of lost reactions.*

*
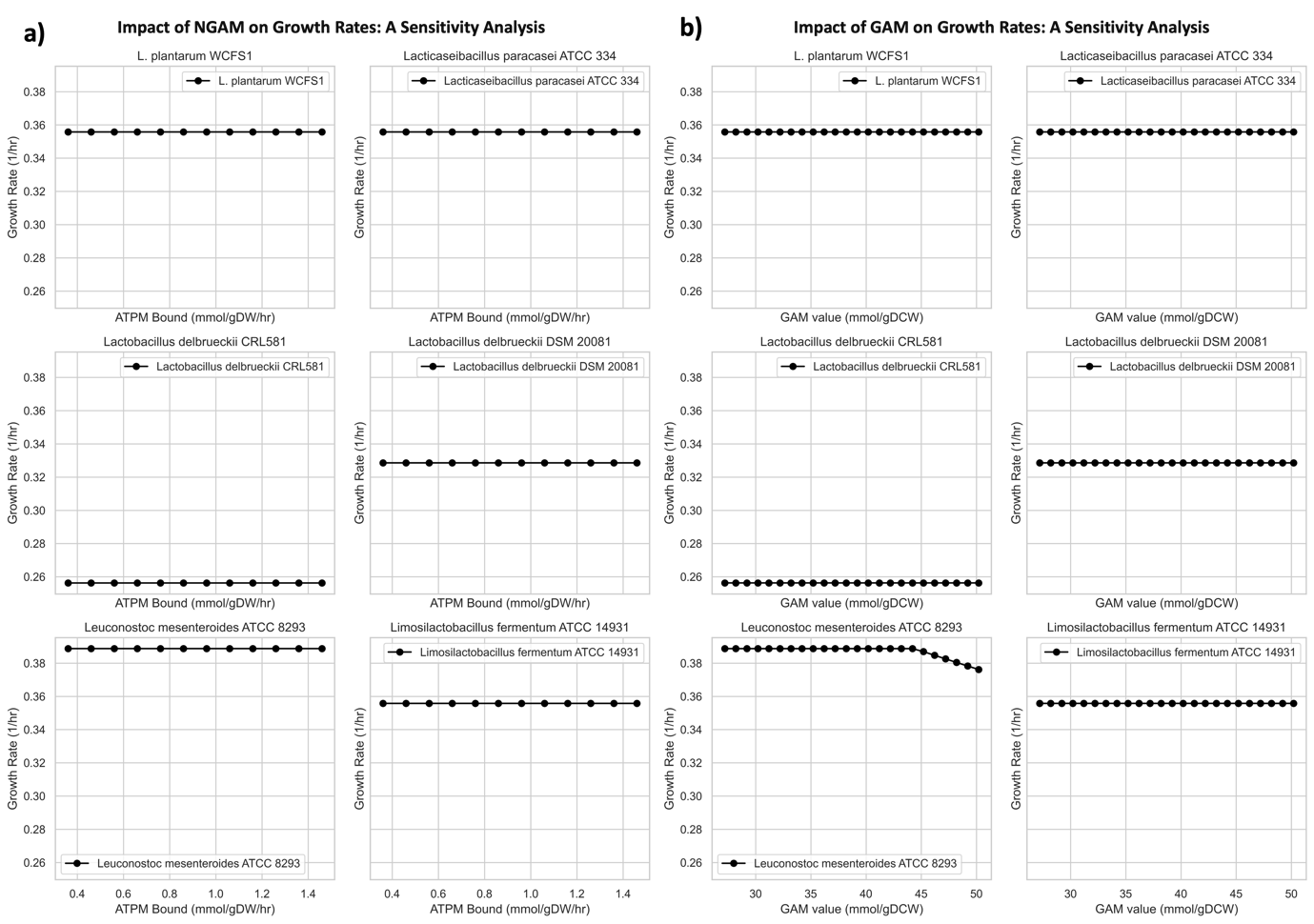
*

***Supplementary Figure 5: Predicted Growth Rate Sensitivity to ATP Maintenance Parameters in GEMs****. a) NGAM Sensitivity: Subpanel A examines the effect of Non-Growth-Associated Maintenance (NGAM) energy on growth rates. The NGAM was varied from 0.36 (lowest published lactobacilli NGAM* ^1^*) to 1.52 mmol/gDCW/h (highest published  lactobacilli NGAM* ^2^*), demonstrating no observable impact on the growth rates of the strains tested. b) GAM Sensitivity: Subpanel B assesses the sensitivity of Growth-Associated Maintenance (GAM) energy on growth rates. GAM values were altered between 10.2 (lowest published lactobacilli GAM* ^3^*) and 49.7 mmol/gDCW/h (highest published lactobacilli GAM* ^4^*) . Consistent growth rates were observed for all strains except for L. mesenteroides ATCC 8293, which showed a reduction in growth rate at GAM levels above 45 mmol/gDCW/h. Benchmark values for GAM and NGAM in the context of this study are set at 27.2 mmol/gDCW/h and 1 mmol/gDCW/h, respectively.*

*
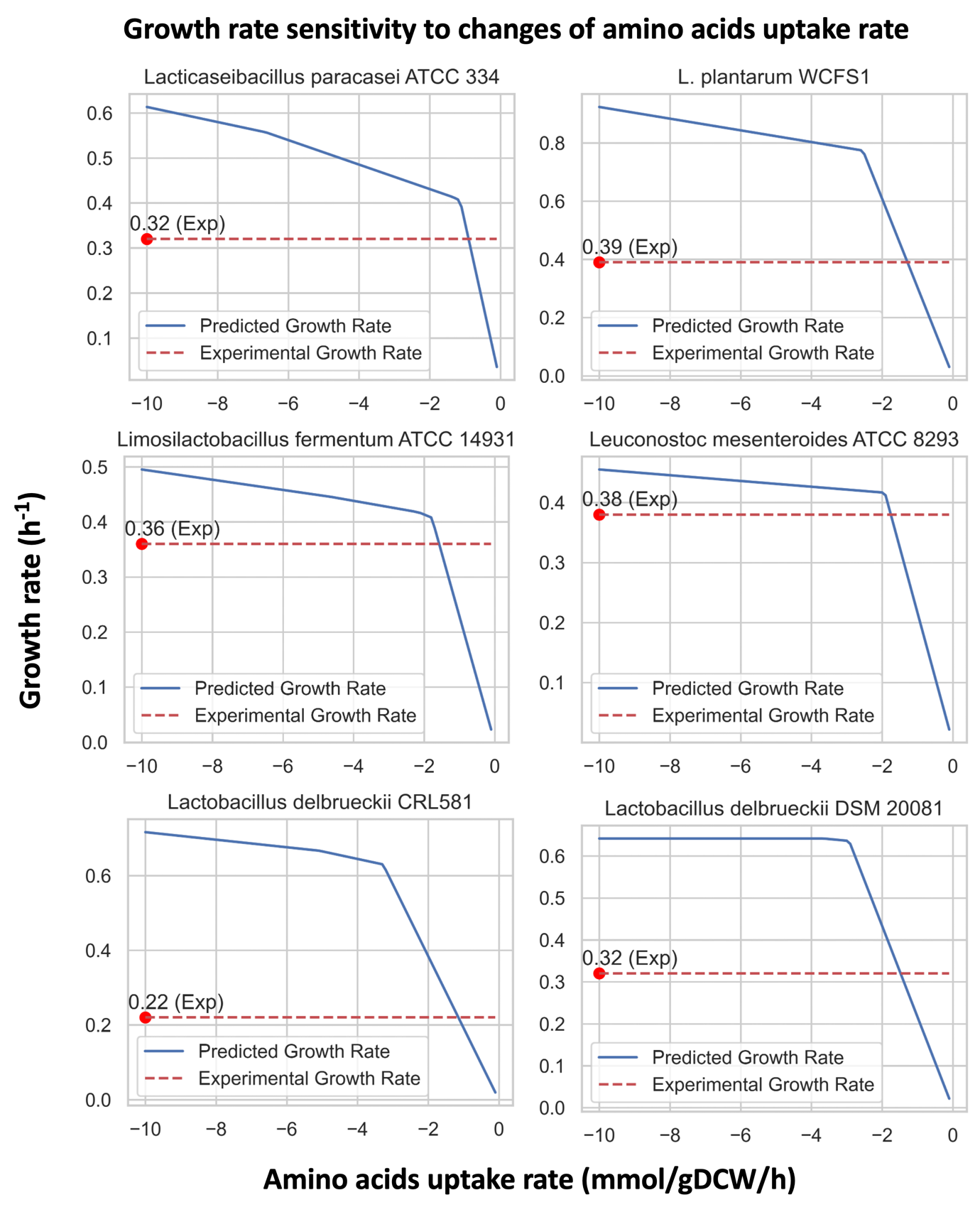
*

***Supplementary Figure 6. Sensitivity Analysis of Predicted Growth Rates to Amino Acid Uptake Rates on Chemically Defined Media (CDM)*** *Each subpanel corresponds to a distinct strain with known growth rates and presents the outcomes of a sensitivity analysis where the lower bounds of amino acid uptake rates were varied incrementally from 0.1 to 10 mmol/gDCW/h. The analysis delineates the relationship between amino acid uptake rates and predicted growth rates, highlighting the degree of sensitivity across the specified range. A pronounced sensitivity is observed in the lower range of 0.1 to 2 mmol/gDCW/h, beyond which, from 2 to 10 mmol/gDCW/h, the growth rates demonstrate reduced sensitivity to changes in uptake rates.*


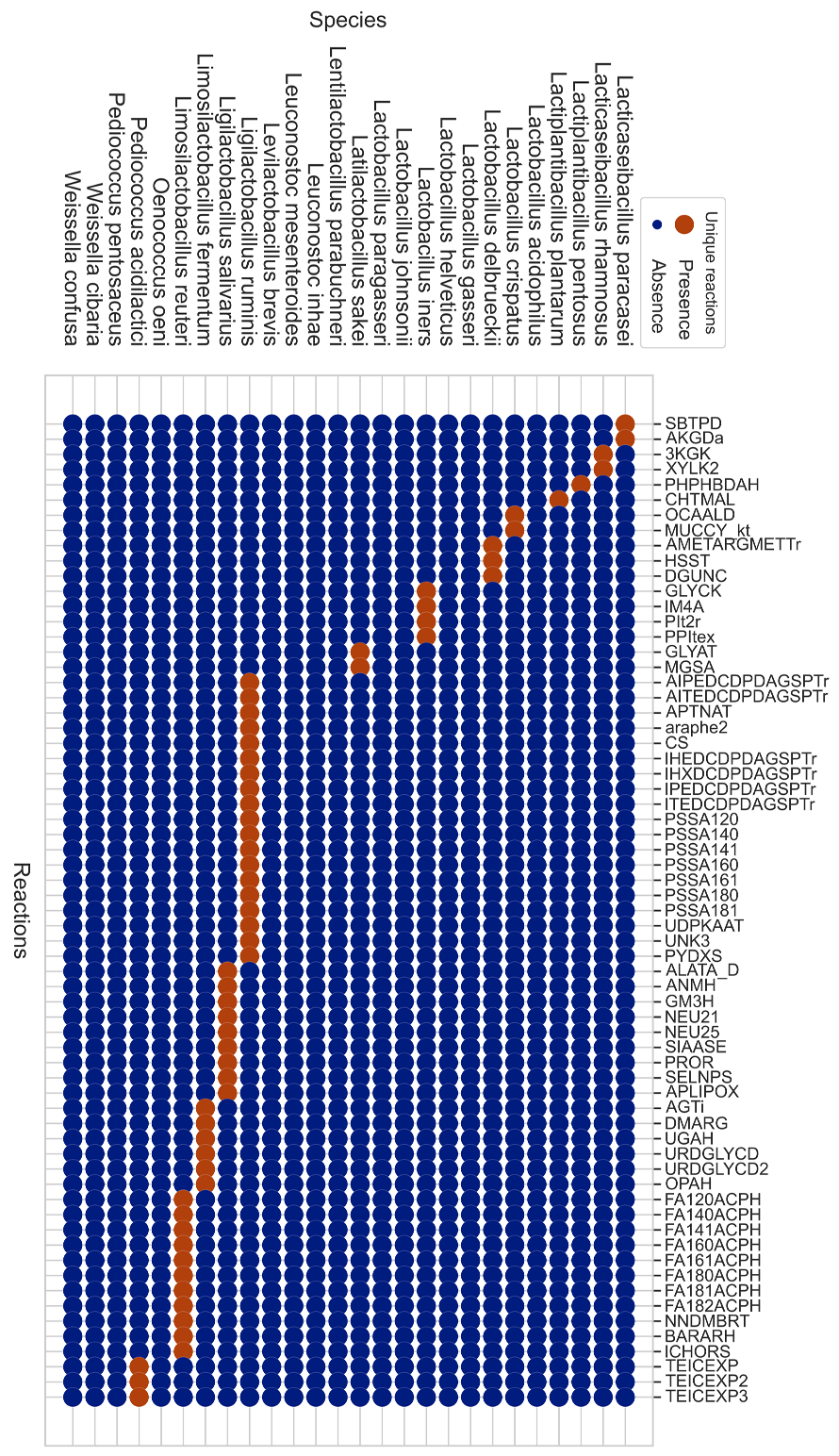


***Supplementary Figure 7: Lactobacillaceae unique reactions.*** *The scatter plot shows the distribution of unique reactions across 26 species of Lactobacillaceae PanGEM, with species-specific (unique) reactions represented by brown dots. L. ruminis stands out as having the highest number of unique reactions (19) among the species examined, while L. reuteri, L. salivarius, and L. fermentum follow with the next highest number of unique reactions. The scatter plot highlights the unique metabolic capabilities of Lactobacillaceae. Lactobacillus reuteri, Lactobacillus salivarius, and Lactobacillus fermentum are commonly found in the human gastrointestinal tract and oral cavity, as well as in other mammalian species. This would suggest that the acquisition of new metabolic capabilities may be an important mechanism for bacteria to adapt and persist in complex environments, such as the human gut. Therefore, the high number of unique reactions observed in these Lactobacillus species may be indicative of their ability to acquire and integrate new metabolic functions from other members of the gut microbiota.*


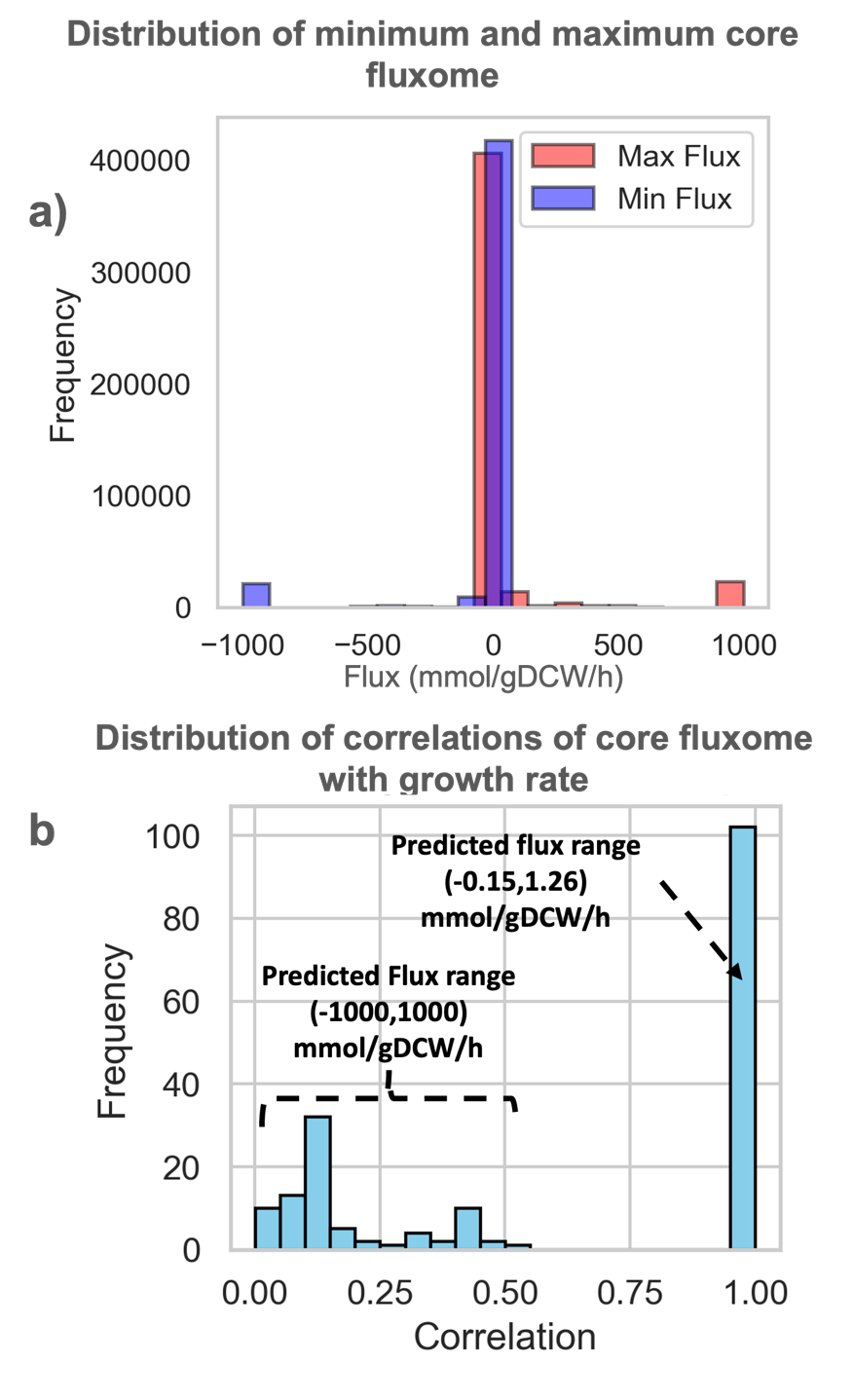


***Supplementary figure 8. Analysis of the Corefluxome in Lactobacillaceae Pan-Genome Models.*** *(a) Illustrates the range of maximum and minimum fluxes observed for core reactions within the Lactobacillaceae pan-genome metabolic models (panGEM). The accompanying histogram reveals that a majority of these reactions exhibit a limited flux variability, suggesting a relative inflexibility of the corefluxome compared to other metabolic reactions. This indicates a constrained adaptability in these core reactions under varying physiological conditions. (b) Presents the correlation between the corefluxome's activity and the growth rates across different strains. Specifically, 74 core reactions were identified to have a perfect correlation coefficient (r=1) with strain growth rates, indicating a direct relationship where strains demonstrating higher fluxes in these reactions tend to exhibit increased growth rates. Furthermore, the annotated flux ranges for these highly correlated reactions are notably narrower, highlighting their essential role and lack of flexibility in the metabolic network, which likely reflects their critical importance for cellular physiology and growth efficiency.*


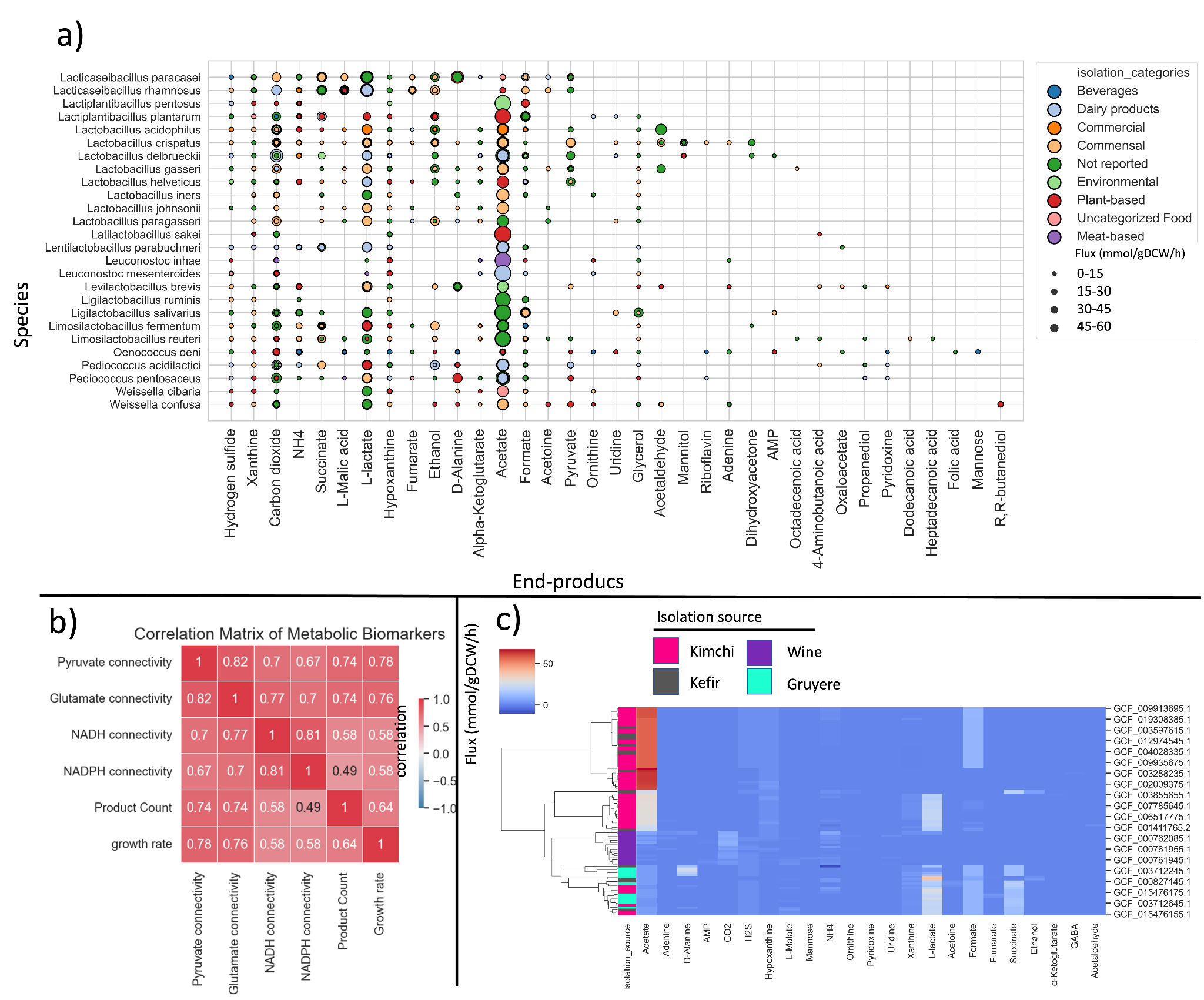


***Supplementary Figure 9: by-product formation prediction****.* ***a)*** *by-product formation across Lactobacillaceae dispersed over isolation categories. Isolation categories are color coded and dot size encodes the production rate of by-products (mmol/gDCW/h).* ***b)*** *Heatmap shows correlations between NADH connectivity, NADPH connectivity, Pyruvate connectivity, Glutamate connectivity, growth rate, and number of by-products across 2446 Lactobacillaceae strains. Pyruvate and Glutamate connectivity show a strong positive correlation (r=0.74) with the number of by-products produced, highlighting the importance of these metabolites in determining the metabolic diversity of Lactobacillaceae.* ***c)*** *Cluster map depicts product formation rate predicted by Lactobacillaceae PanGEM for kimchi, gruyere, and red wine isolates. The majority of strains show clustering based on their isolation source, indicating that these strains are responsible for different organoleptic features of the by-products. Notably, strains isolated from kimchi exhibit a high production rate of acetate, suggesting that the metabolic activity of these strains contributes to the distinctive flavor and aroma of kimchi. These findings highlight the importance of strain selection in the production of fermented foods with desirable organoleptic properties.*


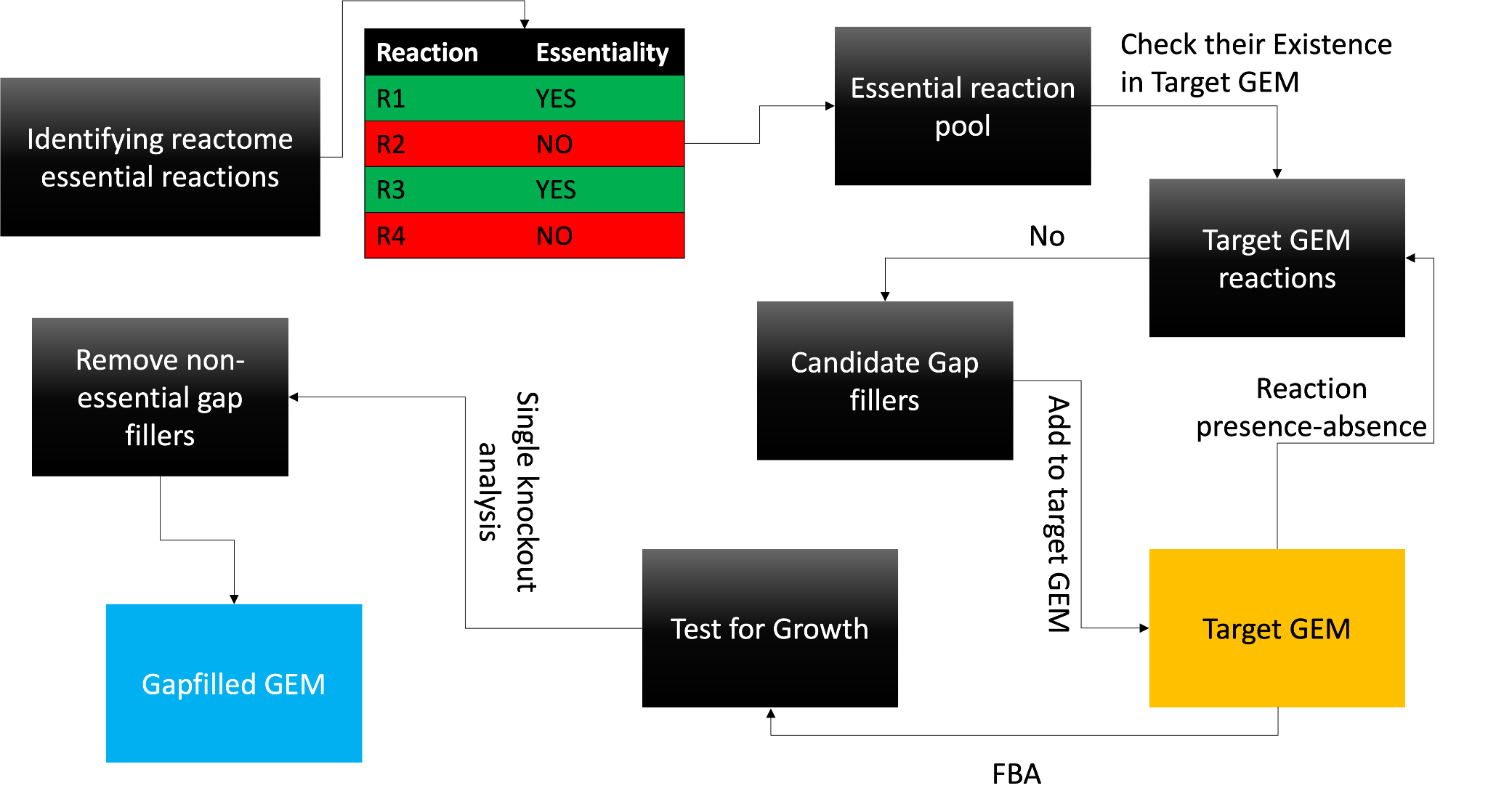


***Supplementary Figure 10: Schematic diagram of the gapfilling procedures used for the reconstruction of 2446 GEMs****. The gapfilling process involved identifying the essential reactions in a reactome and constructing an Essential Reaction Pool (ERP). The target GEM was then checked for the presence of these essential reactions. If any essential reactions were missing, they were added to the target GEM. After gapfilling, growth simulations were performed, and non-essential gapfillers were removed using single knockout simulations. This gapfilling approach enabled the reconstruction of comprehensive and accurate GEMs for a large number of Lactobacillaceae strains, facilitating in-depth metabolic analysis and optimization of these strains for industrial applications.*


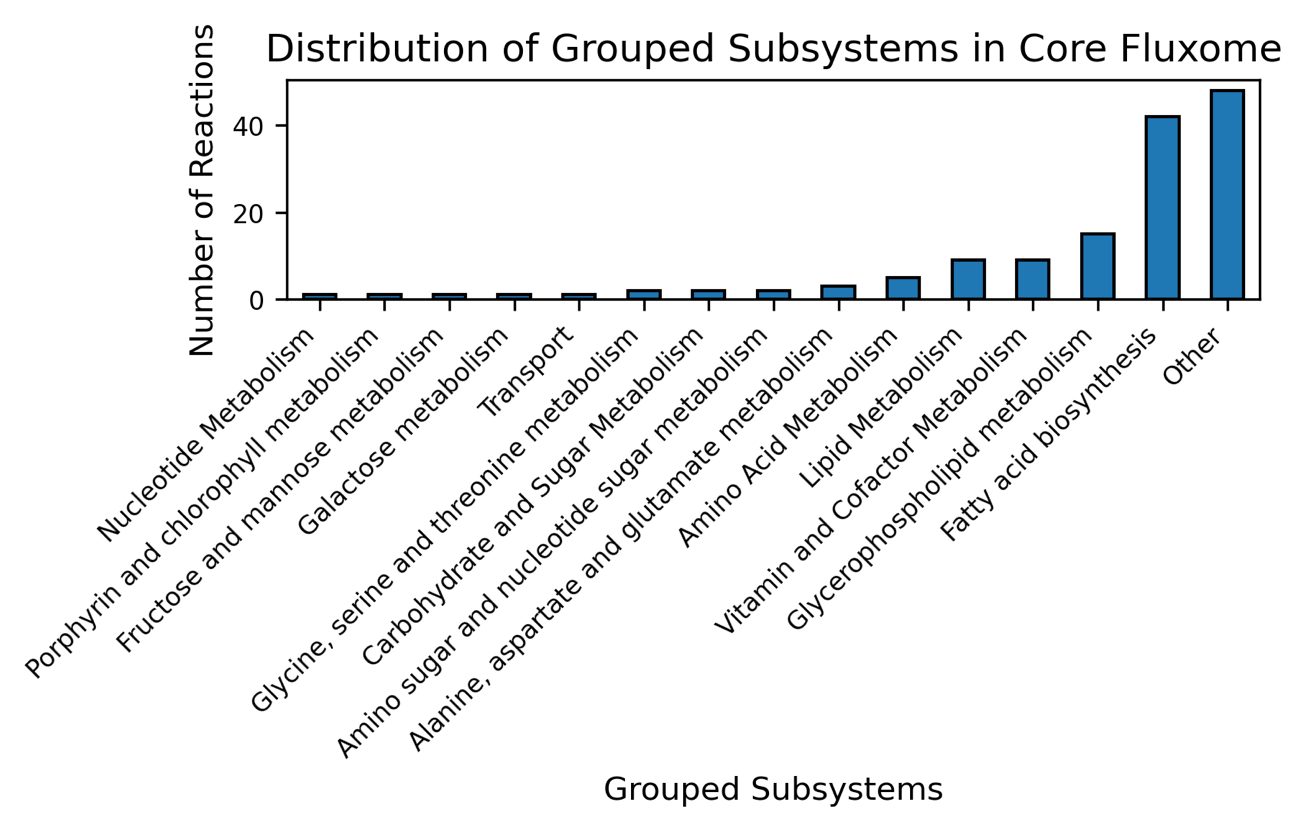


***Supplementary Figure 11: Distribution of essential reactions across metabolic pathways****. The bar chart provides an insight into the metabolic pathways from which the 142 core fluxome originate. Each bar represents a grouped subsystem or pathway, with its height indicating the number of reactions associated with that particular pathway.*

*
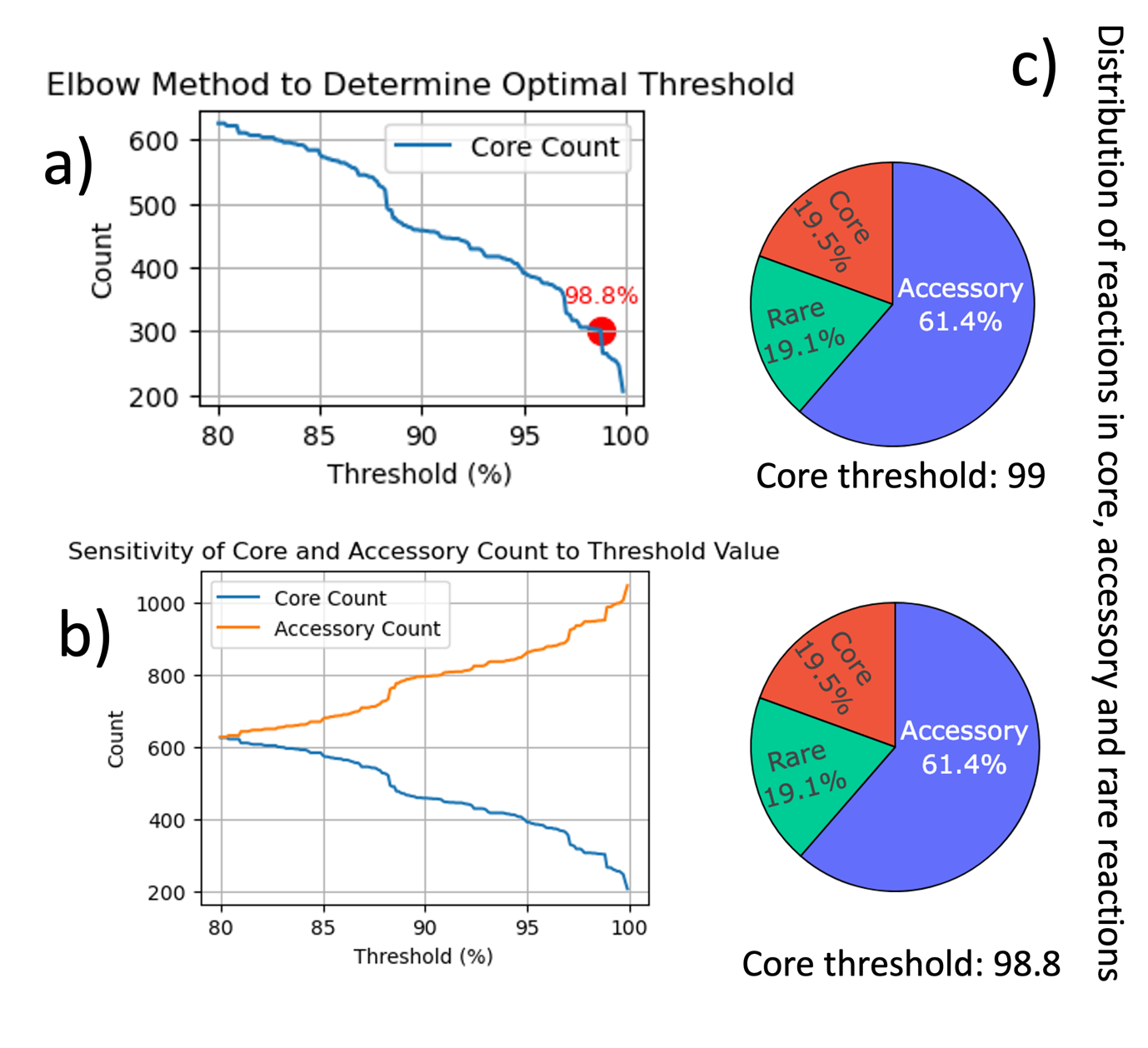
*

***Supplementary Figure 12: The sensitivity of categorizing reactions as core, accessory, or rare within the reactome relative to the threshold values designated for these classifications.*** *a) Sensitivity Analysis of Core Reaction Counts Across Varying Thresholds. This plot illustrates the relationship between different threshold percentages (ranging from 80% to100%) and the corresponding count of reactions classified as core. The red dot marks the optimal threshold, identified at approximately 98.8%, where the rate of change in core reaction counts shows a significant shift. This 'elbow point' is indicative of an optimal balance between inclusivity and exclusivity in defining core reactions within the dataset. b) Variation in Core and Accessory Reaction Counts with Different Threshold Percentages. This plot displays how the counts of reactions categorized as core and accessory fluctuate across a range of threshold percentages (from 80% to 100%). Each line represents the count of reactions falling into the respective category at a given threshold value, highlighting the sensitivity of reaction classification to the chosen threshold. The graph demonstrates the inverse relationship between the core and accessory reaction counts, illustrating the trade-off involved in defining the threshold for core reaction inclusion in the pan-reactome analysis. c) distribution of reactions in ractome categories, pie chart shows the percentage of core, accessory and rare reactions for applied thresholds in this study, results show no changes in the percentages of categories indicating high percentage of accessory and low percentage of rare and core reactome in both scenarios.*

*
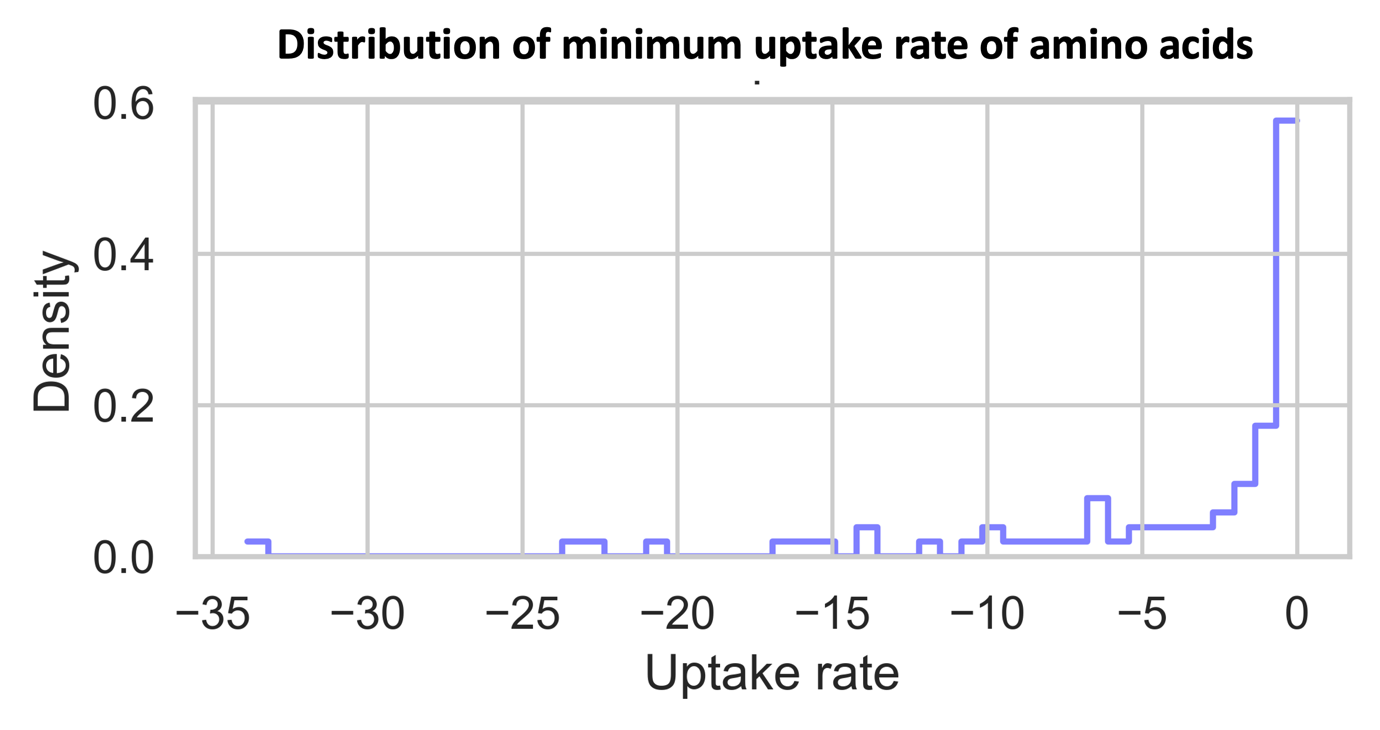
*

***Supplementary Figure 13: Distribution of Amino Acid Uptake Rates as Determined by Flux Variability Analysis (FVA)****. Histogram represents the calculated minimum uptake rates for amino acid exchange reactions, as determined by FVA across the studied conditions. The data illustrates a predominant density of amino acid exchange reactions with minimum uptake rates below the threshold of 1 mmol/gDCW/h. Based on this distribution, a uniform lower bound of 1 mmol/gDCW/h for amino acid exchange reactions has been established to model growth on Chemically Defined Media (CDM) consistently throughout this study(See supplementary Table 3).*

**Table. 1. Global market size of *Lactobacillaceae-related* products**

| **Product​​** | **Market size (billions of U.S. dollars)​​** | **Reference​​** |
| --- | --- | --- |
| **Yogurt​​** | **167​​** | ^8^ |
| **Cheese​​** | **154.8​​** | ^9^ |
| **Butter​​** | **51.49​​** | ^10^ |
| **Sour cream​​** | **1.7​​** | ^11^ |
| **Kefir​​** | **1.3​​** | ^12^ |
| **Soy sauce​​** | **40.63​​** | ^13^ |
| **Red wine​​** | **182.0​​** | ^14^ |
| **Miso​​** | **107.4​​** | ^15^ |
| **Tempeh​​** | **4.7​​** | ^16^ |
| **Kimchi​​** | **3.3​​** | ^17^ |
| **Sauerkrauts​​** | **10.4​​** | ^18^ |
| **Pickles​​** | **11.1​​** | ^19^ |
| **Kombucha​​** | **2.64​​** | ^20^ |
| **Sourdough​​** | **3.13​​** | ^21^ |
| **Vinegar​​** | **6.4​​** | ^22^ |
| **Pepperoni​​** | **2​​** | ^23^ |
| **Salami​​** | **7.5​​** | ^24^ |
| **Fermented fish​​** | **66.9​​** | ^25^ |
| **Chocolate​​** | **113.16​​** | ^26^ |
| **Probiotic Supplements​​** | **6.65​​** | **​​**^27^ |
| **Whey protein powder​​** | **12.41​​** | ^28^ |
| **Lactic acid​​** | **3.1​​** | ^29^ |
| **Hot Sauce​​** | **2.75​​** | ^30^ |
| **Feed probiotics​​** | **4.8​​** | **​​**^31^ |
| **Total market size​​** | **938​​** | **​​** |

***Table. 2. Exchange reaction constraints for CDM simulation by FBA/FVA******

| *Exchange Reaction* | *Lower Bound (mmol/gDCW/h)* | *Compound Name* |
| --- | --- | --- |
| *Carbon source* | | |
| *EX_glc_D_e* | *-25,2* | *D-Glucose* |
| *EX_ac_e* | *-1* | *Acetate* |
| *EX_cit_e* | *-1* | *Citrate* |
| *Amino acids* | | |
| *EX_arg_L_e* | *-1* | *L-Arginine* |
| *EX_cys_L_e* | *-1* | *L-Cysteine* |
| *EX_glu_L_e* | *-2* | *L-Glutamate* |
| *EX_ile_L_e* | *-1* | *L-Isoleucine* |
| *EX_leu_L_e* | *-1* | *L-Leucine* |
| *EX_met_L_e* | *-1* | *L-Methionine* |
| *EX_tyr_L_e* | *-1* | *L-Tyrosine* |
| *EX_phe_L_e* | *-1* | *L-Phenylalanine* |
| *EX_thr_L_e* | *-1* | *L-Threonine* |
| *EX_val_L_e* | *-1* | *L-Valine* |
| *EX_gly_e* | *-1* | *Glycine* |
| *EX_ala_L_e* | *-1* | *L-Alanine* |
| *EX_asp_L_e* | *-2* | *L-Aspartate* |
| *EX_his_L_e* | *-1* | *L-Histidine* |
| *EX_lys_L_e* | *-1* | *L-Lysine* |
| *EX_pro_L_e* | *-1* | *L-Proline* |
| *EX_ser_L_e* | *-1* | *L-Serine* |
| *EX_trp_L_e* | *-1* | *L-Tryptophan* |
| *Nucleotides* | | |
| *EX_ura_e* | *-1* | *Uridine* |
| *EX_gua_e* | *-1* | *Guanosine* |
| *EX_ins_e* | *-1* | *Inosine* |
| *EX_ade_e* | *-1* | *Adenosine* |
| *EX_xan_e* | *-1* | *Xanthine* |
| *EX_orot_e* | *-1* | *Orotate* |
| *Vitamines* | | |
| *EX_btn_e* | *-1* | *Biotin* |
| *EX_pnto_R_e* | *-1* | *Pantothenate* |
| *EX_thm_e* | *-1* | *Thiamine* |
| *EX_pydam_e* | *-1* | *Pyridoxamine* |
| *EX_pydxn_e* | *-1* | *Pyridoxine* |
| *EX_ribflv_e* | *-1* | *Riboflavin* |
| *EX_fol_e* | *-1* | *Folate* |
| *EX_ascb_L_e* | *-1* | *L-Ascorbate* |
| *EX_4abz_e* | *-1* | *4-Aminobenzoate* |
| *EX_nac_e* | *-1* | *N-Acetyl-D-glucosamine* |
| *EX_thymd_e* | *-1* | *Thymidine* |

** The constraints applied to the exchange reactions for Flux Balance Analysis (FBA) and Flux Variability Analysis (FVA) were kept consistent throughout the entire study.*

*In the determination of the lower bound for the glucose exchange reaction, the glucose uptake rate was carefully selected based on recent experimental findings for Lactobacillus reuteri JCM 1112* ^3^*. This approach ensures the adoption of a realistic glucose uptake rate, critical for the accuracy of our metabolic model simulations. For amino acids, a maximum uptake rate was established at a threshold of 1. This decision stems from comprehensive analyses utilizing Flux Variability Analysis (FVA) on Genome-Scale Metabolic Models (GEMs). These models were evaluated under conditions of known growth rates in Chemically Defined Media (CDM). By fixing the growth rates of these GEMs and computing the minimum and maximum uptake rates for amino acids while keeping their exchange reactions unbounded, it was observed that the uptake rates for most amino acids did not exceed the threshold of 1. Consequently, this value was set as the lower bound for the exchange reactions of amino acids in our study. It is noteworthy that Glutamine and Asparagine are not included in the CDM formulation. As a result, the lower bounds for the exchange reactions of Glutamate and Aspartate were adjusted to twice their initial values. This modification is essential for enabling the metabolic models to convert these amino acids at rates that are meaningful from a biological perspective.*

*Table.3 calculated mean NGAM based on published LAB GEM’s NGAM*

| ***Species*** | ***NGAM Value*** | ***Reference*** |
| --- | --- | --- |
| *L. plantarum WCFS1* | *0.36* | ^4^ |
| *L. lactis MG1363* | *0.92* | ^5^ |
| *L. lactis ssp. lactis IL1403* | *1* | ^5^ |
| *L. casei LC2W* | *1.52* | ^4^ |
| *L. lactis FD1 72* | *1* | ^5^ |
| *L. acidophilus La-14* | *1.48* | ^6^ |
| *L. mesenteroides* | *1.1* | ^7^ |
| *Mean NGAM* | *1* |  |

*Non-Growth Associated Maintenance (NGAM) was calculated based on mean NGAM of available GEMs of Lactic Acid Bacteria (LAB)*

**Supplementary Note 1**

A species-specific reactome analysis was performed to understand metabolic conserveness among strains of each species. For this goal, core, accessory, and rare reactomes were calculated for each species based on intra-species reactions commonality. The highest percentage of rare reactome was found for *L. crispatus, L. mesentroides, O. onei,* and *L. fermentum.* On the contrary, *L. iners, L. ruminis*, and *L. acidophilus* had the lowest percentage of rare reactome. Also, the highest percentage of accessory reactome could be found in *L. plantarum, L. helveticus,* and *P. acidilactici. In contrast,* the lowest percentage of accessory could be found in *L. acidophilus, L. iners,* and *L. parabuchneri.* Core reactome showed a high percentage in *L. acidophilus, L. iners, L. parabuchneri,* and *L. ruminis,* while *L. plantarum, L. helveticus, L.crispatus and P.acidilactici.* This shows *L. plantarum* has the most metabolically diverse strains across the whole family, while *L. acidophillus* is the most metabolically conserved species.

**Supplementary Note 2**

To reveal additional metabolic similarities and differences of *Lactobacillaceae*, the essentiality of CDM media components for each GEM has been predicted by FBA (Fig.3-e). This analysis showed that *Lactiplantibacillus plantarum,* with nine consistent auxotrophies among all its members and one inconsistent auxotrophy (Cysteine predicted to be essential in some of *Lactiplantibacillus plantarum* but not all), has the lowest number of auxotophies among all *Lactobacillaceae*, despite *Pediococcus acidilactici* and *cibaria* which have the highest number of auxotrophies, 12 consistent auxotrophies were predicted for both species, while cibaria has eight more inconsistent auxotrophies and this number for *acidilactici* predicted to be seven. Also, regarding the highest number of consistent auxotrophies, *Latilactobacillus sakei,* and *Lactobacillus paragasseri,* with 16 consistent auxotrophies among all strains within these two species, have the highest number of consistent auxotrophies (Fig3.e). Also, four amino acids, including Isoleucine, Valine, Phenylalanine, and Tyrosine, were predicted as globally essential for all 2,446 models in *Lactobacillaceae* PanGEM.

**Supplementary Note 3**

Distinct metabolic profiles were observed among the different *Lactobacillaceae* species based on the clustering patterns seen in the PCA plot. These metabolic differences likely indicate adaptations to various ecological niches as the bacteria must obtain the necessary nutrients for survival and growth in their environment. Differences in preferred ecological niches were apparent in the distinct clustering of *Lactobacillus, Lactiplantibacillus*, and *Lacticaseibacillus* species. *Lactobacillus* species are commonly found in the gastrointestinal tracts of humans and animals. In contrast, *Lactiplantibacillus* species thrive in plant environments such as soil, plant surfaces, and fermenting vegetables. *Lacticaseibacillus* species are known to prefer dairy environments, such as milk and cheese. Notably, a separate cluster of *Lactobacillus ruminis* was observed, suggesting adaptation to a specialized ecological niche within the digestive tracts of cattle and other ruminants. The observed clustering patterns suggested a close connection between bacterial species' metabolic diversity and ecological niches. Further understanding of these metabolic differences and ecological niches can provide valuable insights into the functional roles of these bacteria in different environments and their potential biotechnological applications.

Supplementary Note 4.

The selection of 49 reference genomes served as a strategic foundation for compiling an initial set of Gene-Protein-Reaction (GPR) associations to facilitate broad-scale reactome mapping. These genomes were selected based on the availability of semi-curated Genome-scale Metabolic Models (GEMs) in the Virtual Human Metabolism (VHM) database. The purpose was to enable direct comparison with our reactome to identify any missing reactions before the curation stage.

During the curation phase, it became apparent that gene function assignments by the ModelSEED pipeline contained errors, leading to an extended manual curation process. This issue prompted us to re-annotate the genomes with the PROKKA pipeline instead of using RAST, allowing us to re-assign metabolic functions more accurately. This ensured a high-quality basis for subsequent strain-specific reconstructions.

Once we had established our initial GPRs, a bidirectional blast was conducted against all available GEMs in the BiGG database to identify any overlooked reactions. Crucially, to maintain uniformity and avoid discrepancies in naming conventions, we opted not to integrate GPRs directly from the VHM GEMs into our models. Consistency with BiGG models was a priority to ensure the clarity and transferability of our final GEMs.

Further, as detailed in the Methods section, an extensive review was undertaken to identify missing locus tags within the GPRs of each analyzed genome. This review extended beyond the original 49 genomes and encompassed all 2447 genomes . The result was a curated list of missed metabolic genes, for which we then formulated GPRs that were added to the respective GEMs. This comprehensive approach enabled the discovery of metabolic genes that were absent from the initial GPRs and not identifiable through a bidirectional blast with the BiGG database. The reactome was then updated with the final curated GEMs to encompass all initially missing GPRs, ensuring comprehensive coverage.

This meticulous process resulted in the identification of species-specific GPRs—referred to as unique reactions in our manuscript and illustrated in Supplementary Figure 7. Consequently, the resulting reactome model stands as a high-quality, standardized universal reactome for Lactobacilli, poised to enhance uniformity and transferability across different studies. Further elaboration on this procedure has been added to the Methods section for clarity.

Supplementary Note 5.

The methodology for identifying and integrating missing reactions, which their locus tags are present in our reference genomes but not exist in the Reactome, involved a detailed, step-by-step approach outlined below:

1. We compiled locus tags from all 49 reference genomes and cross-referenced them with locus tags in Reactome’s GPRs to identify locus tags present in the genomes but missing in the Reactome.
2. Locus tags without corresponding entries in Reactome were further Assessed, filtering out those associated with non-metabolic functions to refine the list exclusively to metabolic genes.
3. Further filtration was applied to select metabolic genes relevant to the scope of metabolic reconstruction. For example, reactions involving DNA methylation were omitted despite their metabolic nature because they cause mass balance issues within the context of genome-scale metabolic models.
4. The curated list of metabolic genes was then mapped against the KEGG database to collect associated reactions.
5. Identified KEGG reaction IDs were cross-matched with the BiGG database to obtain their curated equivalents. (where KEGG id were not found in BiGG database, all the reaction names associated with the KEGG id were searched in BiGG)
6. For KEGG reactions without corresponding BiGG curated reactions, the metabolites were cataloged and matched to the BiGG database to ensure consistency in metabolite identification, preventing network discrepancies due to varied nomenclature.(where KEGG id was not found in the BiGG database, all the metabolite names associated with the KEGG id were searched in BiGG)
7. Where no BiGG matches were found for KEGG metabolite IDs, the KEGG IDs were used in GPR formulations as metabolite identifiers.
8. By cataloging which KEGG IDs were matched in BiGG, we updated the reactions from KEGG with unidentified BiGG equivalents based on their metabolite IDs.
9. The directionality of these manually curated reactions was verified by checking against ΔG values in the BioCyc database.
10. Newly formulated GPRs were checked for mass and charge balance using check_mass_balance function of cobrapy and were curated accordingly.
11. Finally, the curated reactions were linked to their corresponding locus tags and incorporated into the respective GEMs.

A similar methodology was applied for strain specific GEMs after their draft GEMs were generated automatically, to capture as much as possible metabolic diversity in our panGEM.

**References**

1. Teusink, B. *et al.* Analysis of growth of Lactobacillus plantarum WCFS1 on a complex medium using a genome-scale metabolic model. *J. Biol. Chem.* **281**, 40041–40048 (2006).

2. Xu, N., Liu, J., Ai, L. & Liu, L. Reconstruction and analysis of the genome-scale metabolic model of Lactobacillus casei LC2W. *Gene* **554**, 140–147 (2015).

3. Kristjansdottir, T. *et al.* A metabolic reconstruction of Lactobacillus reuteri JCM 1112 and analysis of its potential as a cell factory. *Microb. Cell Fact.* **18**, 186 (2019).

4. Namrak, T. *et al.* Probing Genome-Scale Model Reveals Metabolic Capability and Essential Nutrients for Growth of Probiotic Limosilactobacillus reuteri KUB-AC5. *Biology*  **11**, (2022).

5. Flahaut, N. A. L. *et al.* Genome-scale metabolic model for Lactococcus lactis MG1363 and its application to the analysis of flavor formation. *Appl. Microbiol. Biotechnol.* **97**, 8729–8739 (2013).

6. Cunha, E., Zeidan, A. & Dias, O. Towards the reconstruction of the genome-scale metabolic model of Lactobacillus acidophilus la-14. in *Advances in Intelligent Systems and Computing* 205–214 (Springer International Publishing, Cham, 2021).

7. Koduru, L. *et al.* Genome-scale modeling and transcriptome analysis of Leuconostoc mesenteroides unravel the redox governed metabolic states in obligate heterofermentative lactic acid bacteria. *Sci. Rep.* **7**, 15721 (2017).

8. Yogurt - worldwide. *Statista* https://www.statista.com/outlook/cmo/food/dairy-products-eggs/yogurt/worldwide.

9. Baron, C. Largest dairy foods processors in North America 2021, based on sales.

10. Straits Research. Butter market, industry analysis, CAGR, top trends and forecasts to 2030.

11. Sour Cream Market 2023. https://www.marketresearchfuture.com/reports/sour-cream-market-6418.

12. Kefir Market. https://www.futuremarketinsights.com/reports/kefir-market.

13. Soy sauce market size, share, trends. https://www.fortunebusinessinsights.com/soy-sauce-market-102857.

14. Red Wine Market. *Allied Market Research* https://www.alliedmarketresearch.com/red-wine-market-A13400.

15. Miso market. https://www.futuremarketinsights.com/reports/miso-market.

16. Tempeh Market. https://www.futuremarketinsights.com/reports/tempeh-market.

17. Kimchi Market size, report, statistics, trends, research, scope, & global analysis by 2029. https://www.databridgemarketresearch.com/reports/global-kimchi-market.

18. Sauerkrauts Market size, share, trends, opportunities & forecast. *Verified Market Research* https://www.verifiedmarketresearch.com/product/sauerkrauts-market/.

19. Research & Markets ltd. Pickles market: Global Industry Trends, Share, Size, Growth, Opportunity and Forecast 2022-2027. https://www.researchandmarkets.com/reports/5578022/pickles-market-global-industry-trends-share.

20. Kombucha market size, share & trends analysis report by product (conventional, hard), by distribution channel (on-trade, off-trade), by region, and segment forecasts, 2022 - 2030. https://www.grandviewresearch.com/industry-analysis/kombucha-market.

21. Sourdough Market - industry analysis and forecast (2022-2029). *MAXIMIZE MARKET RESEARCH* https://www.maximizemarketresearch.com/market-report/global-sourdough-market/67152/ (2020).

22. Vinegar market size, share & trends analysis report by product (balsamic vinegar, Red Wine vinegar), by source, by flavor, by application, by distribution channel, by sales channel (B2B, B2C), by region, and segment forecasts, 2023 - 2030. https://www.grandviewresearch.com/industry-analysis/vinegar-market-report.

23. Pepperoni market. https://www.imarcgroup.com/pepperoni-market.

24. Reshovski, P. Asia Pacific Sausages and Salami Market Size and Trends [Infographic]. https://www.wm-strategy.com/news/asia-pacific-sausages-and-salami-market-trends-and-size (2022).

25. Processed fish market. *Allied Market Research* https://www.alliedmarketresearch.com/processed-fish-market-A16983.

26. Chocolate market size, share & trends analysis report by product (traditional, artificial), by distribution channel (supermarket & hypermarket, convenience store, online), by region, and segment forecasts, 2022 - 2030. https://www.grandviewresearch.com/industry-analysis/chocolate-market.

27. Global Probiotic Dietary Supplements Market Report and Forecast 2023-2028. https://www.expertmarketresearch.com/reports/probiotic-dietary-supplements-market.

28. Precedence Research. Whey protein market size to worth around USD 21.36 bn by 2032. *Precedence Research* https://www.globenewswire.com/en/news-release/2023/01/09/2584952/0/en/Whey-Protein-Market-Size-to-Worth-Around-USD-21-36-Bn-by-2032.html (2023).

29. Lactic acid market size, share & trends analysis report by raw material (sugarcane, corn, cassava), by application (industrial, food & beverages, pharmaceuticals, personal care, polylactic acid), by region, and segment forecasts, 2023 - 2030. https://www.grandviewresearch.com/industry-analysis/lactic-acid-and-poly-lactic-acid-market.

30. Facts & Factors. Demand for Global Hot Sauce Market size to surpass USD 4.91 Billion by 2026, exhibit a CAGR of 7.9%. *Facts & Factors* https://www.globenewswire.com/en/news-release/2022/10/04/2527834/0/en/Demand-for-Global-Hot-Sauce-Market-Size-to-Surpass-USD-4-91-Billion-by-2026-Exhibit-a-CAGR-of-7-9-Hot-Sauce-Industry-Trends-Share-Value-Analysis-Forecast-Report-by-Facts-Factors.html (2022).

31. MarketsandMarkets Research Pvt. Ltd. At CAGR of 8.8% the probiotics in animal feed market to cross $7.3 billion by 2026 globally report by MarketsandMarkets^TM^. *MarketsandMarkets Research Pvt. Ltd* https://www.globenewswire.com/news-release/2023/01/31/2598536/0/en/At-CAGR-of-8-8-The-Probiotics-in-Animal-Feed-Market-to-Cross-7-3-billion-by-2026-Globally-Report-by-MarketsandMarkets.html (2023).
